# A multimodal pipeline for the identification and diagnostic immunoPET validation of hepatocellular carcinoma targets for radiotheranostic development

**DOI:** 10.7150/thno.136906

**Published:** 2026-07-29

**Authors:** Philip Homan, Joon-Yong Chung, Woonghee Lee, Divya Nambiar, Julia Sheehan-Klenk, Hima Makala, Stanley Fayn, Orit Jacobson, Arthur Paden King, Kyungeun Kim, Jeong Won Kim, Sung Ryol Lee, Maggie Cam, Stephen M. Hewitt, Xin Wei Wang, Mitchell Ho, Freddy E. Escorcia

**Affiliations:** 1 Advanced Biomedical Computational Science, Frederick National Laboratory for Cancer Research, Frederick, MD, 21702, USA.; 2 Molecular Imaging Branch, Center for Cancer Research, National Cancer Institute, National Institutes of Health, Bethesda, MD 20892, USA.; 3 Oxford Institute for Radiation Oncology, Department of Oncology, University of Oxford, Oxford Ox3 7DQ, UK.; 4 Department of Pathology, Kangbuk Samsung Hospital, Sungkyunkwan University School of Medicine, Seoul, 03181, Republic of Korea.; 5 Department of Pathology, Kangnam Sacred Heart Hospital, Hallym University College of Medicine, Seoul, 07441, Republic of Korea.; 6 Department of Surgery, Kangbuk Samsung Hospital, Sungkyunkwan University School of Medicine, Seoul, 03181, Republic of Korea.; 7 Center for Collaborative Bioinformatics, National Institutes of Health, Bethesda, MD 20892, USA.; 8 Laboratory of Pathology, Center for Cancer Research, National Cancer Institute, National Institutes of Health, Bethesda, MD 20892, USA.; 9 Laboratory of Human Carcinogenesis, Center for Cancer Research, National Cancer Institute, National Institutes of Health, Bethesda, MD 20892, USA.; 10 Laboratory of Molecular Biology, Center for Cancer Research, National Cancer Institute, National Institutes of Health, Bethesda, MD 20892, USA.; 11 Radiation Oncology Branch, Center for Cancer Research, National Cancer Institute, National Institutes of Health, Bethesda, MD 20892, USA.

**Keywords:** hepatocellular carcinoma, precision oncology, drug development, radiotheranostics, radiopharmaceuticals

## Abstract

**Background:**

Identifying tumor selective targets is critical for the development of precision diagnostic and therapeutic agents in oncology. Despite advances in precision oncology elsewhere, there are no FDA-approved hepatocellular carcinoma (HCC) antigen-selective antibody-drug conjugates or radiopharmaceuticals. This study establishes an integrated workflow to identify HCC-enriched plasma membrane targets and assess their suitability for targeted molecular imaging as foundational candidates for future radiopharmaceutical therapy.

**Methods:**

Bulk RNA sequencing (371 tumors), single cell RNA sequencing (34 HCC cases), and a normal liver dataset were analyzed to identify HCC-enriched plasma membrane targets. Candidate molecules were examined on HCC and normal tissue microarrays (TMAs) and further evaluated in liver cancer cell lines by quantitative PCR, Western blot, and flow cytometry. Selected targets were then tested in mouse models of liver cancer using antibody-based positron emission tomography (immunoPET) to assess *in vivo* target engagement and distribution.

**Results:**

Integrated transcriptomic analysis identified several tumor plasma membrane molecules with strong tumor enrichment, including GPC3, MUC13, TSPAN8, MET, and EGFR. TMAs confirmed prominent membrane expression in HCC with little signal in normal organs. Combinations of four prioritized markers captured up to 88.5% of patient tumors. Antibody-based immunoPET agents directed against prioritized targets demonstrated specific tumor accumulation *in vivo*, and signal intensity correlated with membrane staining by immunohistochemistry. These results highlight the potential of these markers to serve as diagnostic tools and promising candidate platforms for therapeutic development, demonstrating the utility of this approach to identify novel oncology targets.

**Conclusions:**

This study establishes a multimodal framework integrating transcriptomic predictions and experimental protein validation to identify HCC targets for molecular imaging and radiopharmaceutical development. This translational blueprint successfully advances precision diagnostic and theranostic-ready agents for this disease for HCC, and a similar approach may be useful for identifying and validating targets in other malignancies.

## Introduction

Hepatocellular carcinoma (HCC) is the most common cause of liver cancer and accounts for over 500,000 new cases and as many deaths annually worldwide 1. While transplantation and surgical resection can be curative, and several locoregional therapies are available for early-stage disease, most patients present with advanced or metastatic disease. For these patients, systemic agents are indicated 2.

Despite recent advances in precision oncology, the clinical management of HCC remains heavily constrained by the absence of approved systemic options designed to selectively engage tumor-specific cell-surface antigens. Targeted therapies in the form of kinase inhibitors were the first to significantly improve survival for patients with HCC 3-5. More recently, immunotherapy-based regimens have come to the forefront, demonstrating superior outcomes compared to kinase inhibition alone 6, 7. While these agents have fundamentally changed our approach to managing patients with advanced and metastatic HCC, the 5-year overall survival of this cohort is still only about 20%. Furthermore, this class of agents is not truly precision oncology, where a unique feature, such as a novel oncogenic fusion or overexpressed molecule, of the cancer is specifically exploited to yield superior therapeutic benefit, thereby improving overall survival and/or quality of life for patients receiving the novel therapy.

In other malignancies antibody-drug conjugates (ADCs) targeting HER2, TROP2, and Nectin-4 have been successfully tested in clinical studies, yielding regulatory approvals in breast 8, 9, and urothelial cancers 10, respectively. Additionally, targeted antibodies specific to CD20, peptides specific to somatostatin receptor (SSTR), and peptidomimetics specific for prostate specific membrane antigen (PSMA), and small molecules targeting norepinephrine receptors have been coupled to therapeutic radioisotopes that are clinically approved for patients with recurrent B-cell lymphomas 11-13, gastroenteropancreatic neuroendocrine tumors 14, metastatic castration-resistant prostate cancers 15, and paragangliomas 16, respectively.

Some HCC-enriched therapeutic targets, including Glypican-3 (GPC3) and basigin (BSG, CD147), have been explored in preclinical and clinical trials 17-21. Imaging of GPC3 using an antibody-based positron emission tomography agent (immunoPET) proved successful in a clinical trial of patients with HCC, however, the imaging agent was found to not predict for response to kinase inhibition 22. Preclinically, Fu et al. engineered a GPC3-targeted ADC and demonstrated efficacy in murine models 23. We and others have successfully used alpha particle-emitting radiopharmaceutical therapy to treat animals with GPC3-expressing liver cancer xenografts 24-26. Early clinical data using a GPC3-targeted peptide-based PET agent appear promising 27. In China, a radioiodine-labeled CD147-specific antibody fragment, ^131^I-metuximab, is approved for transarterial adjuvant treatment following resection for patients with HCC 21. Because HCC is radiosensitive as it is routinely treated with external beam radiotherapy and transarterial radioembolization, it stands to reason that targeted radiopharmaceutical therapy may represent a viable treatment option for patients with this disease. The successes targeting these highly overrepresented molecules for therapeutic intent suggest that an approach to identify additional targets could yield novel therapeutics and diagnostics, also referred to by the portmanteau “theranostics” or “radiotheranostics” when specifically using radionuclides, for patients 28, 29. Confirming robust *in vivo* target engagement via imaging is critical for radiotheranostic agent translation.

Tumor plasma membrane associated targets are of particular interest for radiotheranostic development because they are easily accessible to parenterally administered agents. To the best of our knowledge, no systematic evaluation of plasma membrane targets has been performed to generate a library of putative targets for HCC. Such an approach may help inform combinatorial treatments and begin to address the known challenges of tumor heterogeneity in the development of therapeutic agents.

Here, we perform *in silico* screens of bulk (The Cancer Genome Atlas, TCGA) and single cell RNA sequencing (scRNAseq) patient datasets to identify a list of plasma membrane molecules that are overexpressed on HCC compared to non-tumor samples. We then assess whether these putative targets are also present on patient tissue microarrays (TMAs) of tumors and normal tissues and evaluate their expression in commercially available liver cancer cell lines by flow cytometry and western blot. Next, we design, synthesize, and successfully test immunoPET agents to select targets and confirm their utility for imaging in several murine models of liver cancer. Together, this work demonstrates proof-of-concept that our target discovery approach can yield highly targeted and tumor-enriched agents possessing strong potential to be engineered for future radiotheranostic translation, as well as immediate diagnostic or therapeutic applications.

## Materials and Methods

### TCGA bulk RNA sequencing analysis

Bulk RNA-seq data and associated clinical information were obtained from TCGA. HTseq expression data for Liver Hepatocellular Carcinoma (LIHC) were obtained from the TCGA-LIHC dataset 30. Data analysis and visualization were performed within the NIH Integrated Data Analysis Portal (NIDAP) using R programs developed on the Foundry platform (Palantir Technologies, Denver, CO, USA). Expression data from TCGA- LIHC generated by HTseq were transformed to Counts Per Million (CPM) counts, and genes with CPM values less than 6 in at least 50 samples were removed. Differentially expressed genes between tumor and normal samples were identified using the voom algorithm 31 from the Limma R package (version 3.40.6) 32. Genes with adjusted *p* value ≤ 0.005 and log_2_ fold change of ≥ 1.0 were considered significantly differentially expressed.

### Single cell RNA sequencing dataset analysis

scRNA-seq data processed using Seurat v4 33. Datasets were filtered to remove low quality cells identified by cell expressing less than 600 genes, total UMIs less than 700, mitochondrial expression greater than 20% and cell complexity (log_10_(Genes/UMI)) less than 0.5. The remaining cells were normalized using SCTransform. Batch correction between samples was corrected using the Seurat anchor-based workflow on 3000 variable features and integration using the Canonical Correlation Analysis (CCA) algorithm. After Integration Principal Component Analysis (PCA) and UMAP dimensionality reduction were performed.

Cell type classification was assigned using SingleR 34 with a reference created from the Human Single Cell Atlas 35. The malignant cell classification from Lichun Ma et al. were identified using InferCNV and described in their paper 36. Cells from GSE149614 that clustered with the previously annotated malignant cells from GSE125449 were also classified as malignant. Malignant cell markers were identified using the MAST 37 in Seurat, comparing malignant cells to all other cell populations. Malignant cell modules were identified using high dimensional weighted gene co-expression network analysis (WGCNA) 38-40, and a heat map was created using correlation of gene expression across all cells. Gene pairs were colored based their co-expression score calculated as shown below:



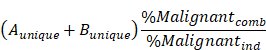



A higher co-expression score for a gene pair indicates that individual genes target different malignant cell populations and combine to target larger percentage of malignant cells compared to an individual gene.

### UniProt membrane-associated gene annotation

Plasma membrane/cell-surface-associated genes were defined using curated UniProt subcellular localization annotations. Genes were retained if their annotations indicated localization to the cell membrane/plasma membrane, cell surface, or related membrane-associated localization consistent with extracellular accessibility (Table S1). This UniProt-defined membrane-associated gene set was used to restrict candidate selection in both the TCGA-LIHC bulk RNA-seq analysis and the integrated single-cell RNA-seq malignant-cell analysis.

### Protein atlas consensus heatmap analysis

Protein Atlas tissue expression was analyzed using the Human Protein Atlas consensus RNA dataset, accessed through the Bioconductor hpar package (hpar v1.50.0; Human Protein Atlas v21.1). Consensus tissue expression values were reported as normalized transcripts per million (nTPM). LIHC tumor expression was added from Human Protein Atlas LIHC cancer sample protein-coding transcripts per million (pTPM) values and restricted to TCGA primary tumor samples.

### Tissue samples

Tissue samples were prospectively collected via surgery from patients who were admitted to the Kangbuk Samsung Hospital or the Kangnam Sacred Heart Hospital between 2010 and 2021. Tumor tissue samples from 165 patients with primary HCC and 165 matched non-adjacent normal epithelium tissues were constructed into TMAs. All the procedures were conducted according to the ethical guidelines of the Declaration of Helsinki, and the study protocol was approved by the Institutional Review Board at Kangbuk Samsung Hospital (IRB No. 2022–10-052-001, Seoul, South Korea) and Kangnam Sacred Heart Hospital (IRB No. HKS2022-12-015, Seoul, South Korea). Additionally, multiple human normal organ TMAs, consisting of 33 organs/99 cores, were purchased from US Biomax, Inc. (Cat. # FDA999L246, Rockville, MD, USA).

### Cell culture

Human hepatocellular carcinoma (HCC) cell lines (Hep3B, SNU182, and SNU449) and a hepatoblastoma cell line (HepG2) were obtained from the American Type Culture Collection (ATCC; Manassas, VA, USA). The Huh7 HCC cell line was obtained from Dr. Mitchell Ho (Bethesda, USA). All cells were tested for mycoplasma using the Mycoplasma Detection Kit (Thermo Fisher Scientific, San Jose, CA, USA) and grown in Dulbecco’s modified Eagle’s medium (DMEM; Thermo Fisher Scientific), Roswell Park Memorial Institute (RPMI) 1640 medium (Thermo Fisher Scientific), or Eagle’s minimal essential medium (EMEM, ATCC) supplemented with 10% fetal bovine serum (Thermo Fisher Scientific) at 37 ℃ under 5% CO_2_ incubator/humidified chamber (PHCbi, Wood Dale, IL, USA).

### Quantitative RT-PCR

Total RNA (1 µg) was extracted using the RNeasy Micro Kit (Qiagen, Valencia, CA, USA) and reverse transcribed with the QuantiTect reverse transcription kit (Qiagen), according to the manufacturer’s protocol. Real-time PCR reactions were performed with 50 ng of cDNA in a total volume of 20 µL, using TaqMan® Universal PCR Master Mix (Applied Biosystems, Foster City, CA, USA) on an ABI 7500 system (Applied Biosystems). Predesigned and labelled primer/probe sets were used for the following genes: *BSG* (Hs00936295_m1), *EGFR* (Hs01076090_m1), *GPC3* (Hs01018936_m1), *TSPAN8* (Hs00610327_m1), *MET* (Hs01565584_m1), *MUC13* (Hs00217230_m1), *ROBO1* (Hs00268049_m1), and *CD24* (Hs02379687_s1). Relative mRNA expression levels were determined using the comparative cycle threshold (2^-ΔΔCt^) method, with *ACTB* as the endogenous control, and were subsequently normalized to the Hep3B cell line.

### Western blot analysis

Cells (5 × 10^6^) were harvested and lysed and extracted with Pierce^TM^ RIPA buffer (Thermo Fisher Scientific) supplemented with Halt^TM^ Protease Phosphatase Inhibitor Cocktail (Thermo Fisher Scientific). Protein concentration was determined using the Pierce^TM^ BCA Protein Assay kit (Thermo Fisher Scientific) according to the manufacturer’s instructions. Proteins (20 - 50 μg) were resolved using 4-12% sodium dodecyl sulphate-polyacrylamide gel electrophoresis (SDS-PAGE) and then transferred onto a nitrocellulose membrane (Thermo Fisher Scientific) using an electric transfer system (Thermo Fisher Scientific). The membranes were incubated overnight at 4 ℃ with primary antibodies: Anti-Basigin/EMMPRIN (#13287), anti-Met/pre-Met (#8198), anti-MUC13 (#44454), and anti-EGF Receptor (#4267), all diluted 1:1000, obtained from Cell Signaling Technology (Danvers, MA, USA); anti-TSPAN8 antibody (ab70007; dilution 1:1000; Abcam, Cambridge, MA, USA); anti-CD24 antibody (SWA11 clone; dilution 1:1000; Cell science, Newburyport, MA, USA); anti-GPC3 antibody (1G12 clone; dilution 1:1000; Cell Marque, Rocklin, CA, USA); and anti-ROBO1 antibody (22D5 clone; dilution 1:1000; Thermo Fisher Scientific). Anti-β-actin (Cell Signaling Technology) was used as the loading control. Subsequently, the membrane was then incubated with horseradish peroxidase-conjugated anti-mouse (#32430) or anti-rabbit (#32460) (Thermo Fisher Scientific) secondary antibodies, and the immunoreactive bands were visualized using Clarity^TM^ Western ECL Substrate (#1705061) or Clarity Max^TM^ ECL Substrate (#1705062) (Bio-Rad, Hercules, CA, USA). The bands were then detected using a ChemiDoc MP imaging system (Bio-Rad).

### Flow cytometry

Approximately 5 × 10⁵ HCC (Hep3B, SNU182, SNU449, and Huh7) and hepatoblastoma (HepG2) cells were harvested and washed with ice-cold FACS buffer (1% bovine serum albumin in 1× PBS). The cells were incubated with an Fc block (Miltenyi Biotec, cat. no. 13005990, 1:50) on ice for 15 min, followed by incubation with monoclonal antibodies against targets or matching isotype controls (Table S2) for 45 min at 4 °C in FACS buffer. After washing with FACS buffer, corresponding fluorescence-conjugated secondary antibodies (Table S2) were added and incubated for 15 min at 4 °C in the dark. The labeled cells were then washed, and cell-associated fluorescence signals were evaluated using a flow cytometer (Cytoflex, Beckman Coulter, Brea, CA, USA). Data were analyzed using FlowJo software v.10.8.1 (FlowJo LLC, Ashland, OR, USA). In brief, the sequential gating strategy involved an initial FSC-A/SSC-A gate to eliminate debris, followed by an FSC-A/FSC-H gate to isolate intact single cells. Thresholds for positive populations were stringently set according to their corresponding isotype.

### Immunohistochemistry

Immunohistochemistry (IHC) was performed on formalin-fixed, paraffin-embedded human HCC, human normal organ, or murine xenograft tissues using EnVision+ Dual Link System-HRP (DAKO, Carpinteria, CA). A mouse-on-mouse IHC kit (M.O.M. kit, Vector Laboratories, Newark, CA, USA) was used to detect CD24 expression in murine xenograft tissues according to the manufacturer’s protocol. Detailed IHC conditions are outlined in Table S3. The antigen-antibody reaction was visualized with DAB+ (3,3-diaminobenzidine; DAKO) for 10 min. TMA sections were lightly counterstained with Mayer’s hematoxylin and examined under light microscopy. Negative control immunoglobulin G (IgG) was used in place of a primary antibody to evaluate nonspecific staining, and appropriate positive control specimens were included within the TMA. The stained TMA slides were scanned using a NanoZoomer 2.0 HT (Hamamatsu Photonics K.K., Hamamatsu City, Japan) at 20× objective magnification. Digital analysis of the images was performed using Visiopharm Integrator System v6.5.0.2303 (VIS; Visiopharm, Hørsholm, Denmark). Briefly, individual digital images of TMA cores were extracted from the whole slide images. The intensity of brown cytoplasmic and membranous staining for potential theranostic markers was quantified using a predefined algorithm and optimized settings (Figure S1), as previously described 41. The average intensity of staining from the two tumor cores was calculated as the final value for membranous and cytoplasmic expression.

### Murine xenograft model

All animal procedures were conducted in accordance with institutional guidelines under a protocol approved by the Institutional Animal Care and Use Committee at the National Institutes of Health. HepG2, Hep3B, and Huh7 cells (5 × 10^6^ cells per mouse) were subcutaneously inoculated into the right flank of female athymic nu/nu mice (7–10 weeks old; Charles River Laboratories, Wilmington, MA, USA). Tumors were allowed to grow to approximately 200 mm³ prior to immunoPET studies.

### Radioconjugate synthesis and quality assurance

A panel of antibodies targeting human tumor-associated antigens was used for radiolabeling. These included codrituzumab (GC33; anti-GPC3 IgG1, Chugai Pharmaceutical Co., Ltd, Tokyo, Japan), panitumumab (anti-EGFR IgG2κ, Amgen, Inc., Thousand Oaks, CA, USA), α-hTSPAN8 (rat anti-human IgG2B, #MAB4734, Bio-Techne, Minneapolis, MN, USA), onartuzumab (monovalent humanized anti-MET chimeric OA5D5 antibody, Genentech, San Francisco, CA, USA), α-hCD24 (anti-human CD24, a proprietary, high-affinity humanized monoclonal IgG1 antibody; supplier undisclosed due to ongoing intellectual property and patent evaluations), and an in-house anti-CD147 nanobody (DMN1) 42. Antibodies were conjugated with a 2.5–3-fold molar excess of *p*-isothiocyanatobenzyl-desferrioxamine (DFO-Bz-NCS; Macrocyclics, Inc.) using an established protocol 43. Radiolabeling with zirconium-89 ([^89^Zr]Zr-oxalate; NIH Cyclotron Facility, Bethesda, MD, USA) was carried out by combining 37–130 MBq of [^89^Zr]Zr-oxalate (diluted in 0.5 M HEPES, pH 7.1–7.3) with DFO-antibody conjugates and 10 µL of 2,5-dihydroxybenzoic acid (5 mg mL⁻¹), followed by pH adjustment using 2 M Na₂CO₃. Reactions were incubated at 37 °C for 1 h and challenged with 10 µL of 100 mM EDTA to verify complex stability. Radioconjugates were purified using PD-10 columns (Cytiva, Marlborough, MA, USA) with phosphate-buffered saline (PBS). Radiochemical purity and labeling efficiency were assessed by radio-instant thin-layer chromatography (radio-iTLC) using iTLC-SG strips (Varian, Lake Forest, CA, USA) with 50 mM EDTA in 100 mM ammonium acetate (pH 5.5) as the mobile phase and analyzed on an AR-2000 radio-TLC scanner (Eckert & Ziegler, Wilmington, MA, USA). All zirconium-89 radiotracers were obtained with high radiochemical purities (> 98%), with individualized specific activities and molar activities thoroughly documented for each specific conjugate, including [^89^Zr]Zr-GC33, [^89^Zr]Zr-Pani, [^89^Zr]Zr-DFO-α-hCD24, and [^89^Zr]Zr-DFO-α-hTSPAN8; a comprehensive compilation of these batch-specific quality control parameters and sample sizes (n) is summarized in Table S4.

For fluorine-18 radiolabeling, the DMN1 nanobody was labelled via a two-step method as described previously 44. [^18^F]FPy-TFP was synthesized on a Sep-Pak cartridge at ambient temperature and reacted directly with DMN1 in phosphate buffer (pH 9.2) at 37 °C for 15–20 min. The crude product was purified using a PD-10 column with saline. The final [^18^F]FPy-DMN1 product showed > 99% radiochemical purity with no detectable aggregation, confirmed by analytical size-exclusion HPLC (TSKgel SuperSW3000, Tosoh Bioscience, King of Prussia, PA, USA). The mobile phase comprised 0.1 M sodium phosphate, 0.1 M sodium sulfate, 0.05% sodium azide, and 10% isopropanol (pH 6.8), run at 0.35 mL min⁻¹. Radiochemical yields were 12–18% (decay-corrected, n = 5), with specific activities ranging from 247 to 740 GBq/mg (corresponding to molar activities of 4 to 11 TBq/µmol). Total synthesis time was approximately 45 min.

### *In vivo* molecular imaging

Mice bearing subcutaneous tumors were intravenously administered 3.7–7.4 MBq (5–10 μg) of ^89^Zr-labeled conjugates or [^18^F]FPy-DMN1 (for CD147 targeting) via tail vein injection. ImmunoPET imaging was performed at highly differentiated, target-specific time points: tailored to the divergent *in vivo* pharmacokinetics and biological clearance profiles of the respective vehicles; a rapid 2 h post-injection scan for the small-molecular-weight [^18^F]FPy-DMN1 nanobody to leverage its fast blood clearance, versus prolonged intervals of 72 h for GPC3 (HepG2 and Hep3B), EGFR (Huh7 and Hep3B), MET (HepG2), and CD24 (Hep3B), and up to 144 h for TSPAN8 (Huh7, Hep3B) to allow the full-length IgG antibodies to clear background tissue and achieve optimal contrast. GPC3 and EGFR imaging was conducted on a BioPET/CT scanner (BioScan Inc., Washington, DC, USA), whereas TSPAN8, MET, CD24, and CD147 imaging used an MRS*PET/CT 120 scanner (MR Solutions, Guildford, UK). Data acquisition employed VISTA (Sedecal, Madrid, Spain) and Preclinical Scan (v4.2.4.0) software (version 4.2.4.0, MR Solutions). Mice were anesthetized with 2% isoflurane, and static PET scans (10–20 min) were acquired, followed by CT for attenuation correction and co-registration. PET data were reconstructed using 3D OSEM and normalized, decay-corrected, and dead-time–corrected. Images were analyzed using MIM software (version 7.2.7, MIM Software Inc., Beachwood, OH, USA).

### Statistical analysis

Data were analyzed using SPSS Statistics for Windows, version 29 (IBM Corp., Armonk, NY, USA), and Prism 10.1.2 (GraphPad Software Inc., San Diego, CA, USA). Differential expression of tumor-associated malignant-cell-enriched membrane-associated markers was assessed with two-tailed paired t-tests. Survival was evaluated using Kaplan–Meier curves and compared by log-rank tests. Combinational expression patterns were visualized with pie charts. For RNA-seq data, we quantified the proportions of malignant cells co-expressing gene pairs or expressing either gene alone. Independent predictors of survival were identified using Cox proportional hazards regression. Statistical significance was defined as *p* < 0.050. To quantify the synergistic advantage of dual-marker combinations over individual targets, a combinatorial co-expression score was mathematically defined for every possible gene pair. For any given pair of Marker A and Marker B, the co-expression score represents the absolute difference between the total percentage of malignant cells expressing either marker A or marker B and the percentage coverage achieved by the single best-performing marker within that pair. Consequently, a score of zero indicates that the expression pattern of the second marker is entirely redundant and completely nested within the first, whereas higher positive scores reflect significant complementary expression profiles that capture independent sub-populations of malignant cells, thereby maximizing cumulative patient coverage.

## Results

### Bulk and single cell RNAseq analysis from patients with HCC identify differentially expressed plasma membrane-associated genes

**Figure 1:**
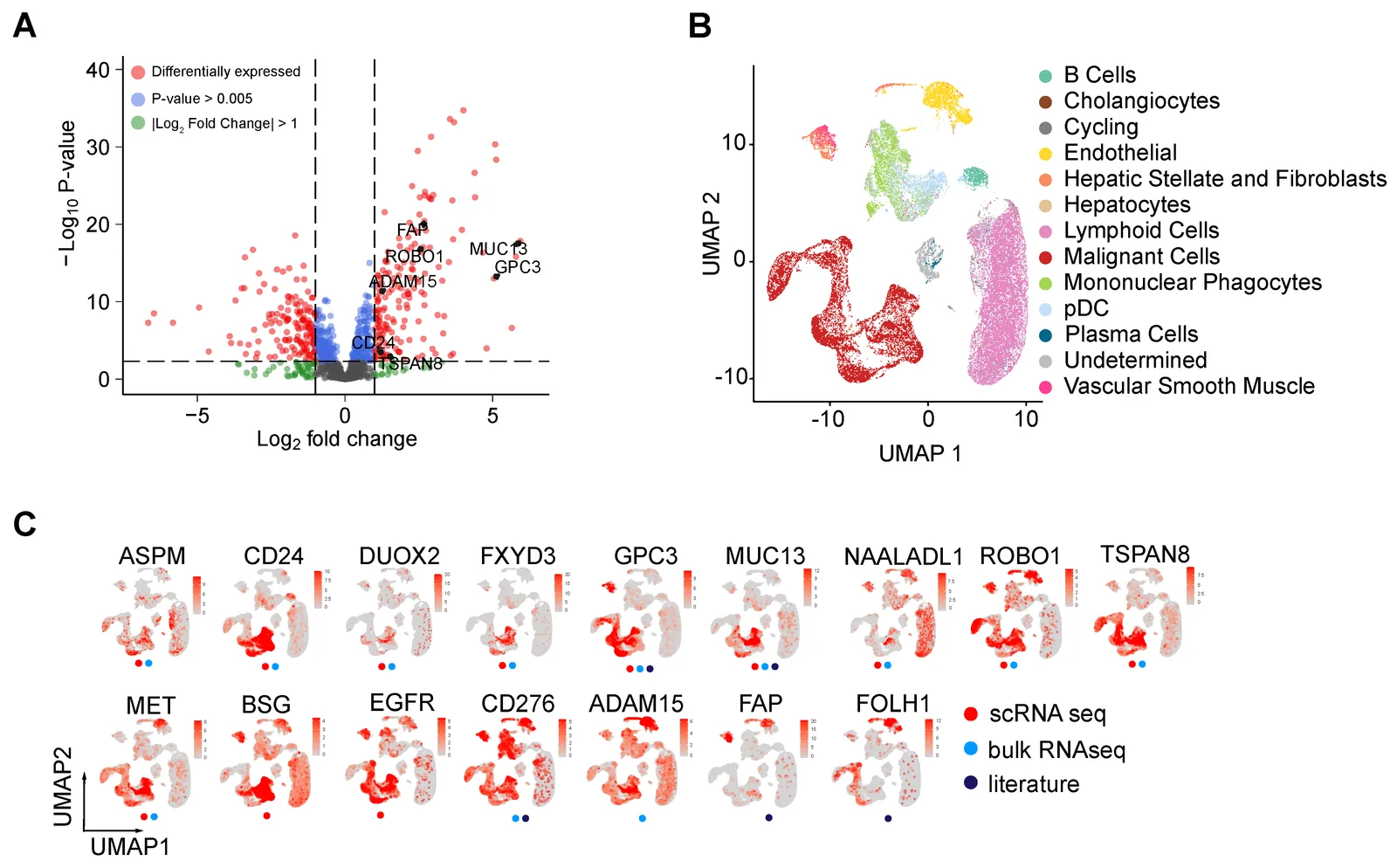
** Identification of overexpressed genes in HCC. (A)** Volcano plot of differentially expressed genes (tumor vs. normal) from TCGA-LIHC RNA-seq expression data (n = 371 tumor samples, n = 50 normal liver samples). Genes colored in red are identified as differentially expressed (*p* value < 0.005 and |Log_2_ Fold Change| > 1). Genes colored in green have a |Log_2_ Fold Change| > 1 and genes colored in blue have *p* value < 0.005. *MUC13*, *GPC3*, *CD24*, *TSPAN8*, *ADAM15*, *ROBO1*, *FAP* are highlighted. **(B)** UMAP of all cells from 41 HCC tumor samples and 8 normal liver samples. Cells are colored by major cell type determined from the Human Single Cell Atlas. Malignant cells (red) were identified from the Lichun Ma *et al.* 36 dataset. **(C)** UMAP plots showing the expression levels of select genes. Plots are marked by red dot if the gene was identified as differentially expressed in the single cell RNA-seq dataset, blue differentially expressed in RNA-seq dataset, or dark blue if identified in the literature.

To identify potential plasma membrane genes associated with liver hepatocellular carcinoma, we compared the RNA-seq, gene expression profiles of LIHC tumor samples from TCGA to normal liver tissue. The dataset consisted of 371 tumor and 50 normal liver samples. PCA was performed to investigate whether tumor and normal liver samples exhibit distinct molecular signatures. The results revealed that tumor and normal liver tissues formed clearly separate clusters, highlighting substantial differences in their molecular profiles (Figure S2A). Differential Expression analysis between Tumor and Normal samples was performed and results were filtered to retain only genes within the membrane-associated gene set defined by UniProt annotations. 45. The analysis identified 156 genes differentially expressed in the tumor samples (Figure 1A).

To further identify high quality cell surface markers directly associated with tumor malignancy from the tumor associated markers, we examined previously published single cell datasets associated with hepatocellular carcinoma. 31 HCC tumor samples published by Lichun Ma *et al.* 36 were integrated with 10 HCC tumor samples and 8 normal liver samples published by Yiming Lu *et al.* 46. After quality control filtering of each sample the integrated dataset contained 102,956 cells. We performed unsupervised clustering and annotated cells using the data from the Liver Single Cell Atlas 35, which contained 28 human liver biopsies from 5 different studies (Figure 1B). Malignant cells were previously identified within tumor liver samples from Lichun Ma *et al.* by inferring large scale chromosomal copy-number variations (CNVs) 36. All malignant cells from this study clustered into a distinct population. Twenty-five percent of the tumor cells from the Yiming Lu *et al.* dataset also clustered within this population and were annotated as malignant. Importantly, normal liver samples from this dataset were included. We found that the relative cell type proportion remained consistent between both datasets (Figure S2B). Furthermore, less than 10% of the normal tissue cells clustered with the malignant cell population (Figure S2C-D).

We then identified genes enriched in malignant cells by comparing the malignant cell population to all other liver cell populations. The resulting differentially expressed genes were filtered to retain only candidates within the UniProt-defined membrane-associated gene set. We identified 63 differentially expressed cell surface markers specific to the malignant cell population. An overlay of gene expression from genes extracted from the current analysis and literature shows that the expression of certain target gene is enriched in malignant cells, though others had broader expression in normal cells (Figure 1C).

We found that 10 genes were classified as both malignant enriched in the single cell samples and tumor enriched from the bulk RNAseq TCGA dataset (Figure S3A). These genes show a high specificity for the malignant cell population (Figure 1C and S3B). *CD24*, *GPC3*, and *TSPAN8* each were expressed in approximately 50 percent of malignant cells.

### Gene expression from Human Protein Atlas helps identify liver specific genes

We further assessed the extent of non-liver tissues expression of the targets identified in the malignant-cell-enriched membrane-associated genes. Human Protein Atlas database (PADB) 47 expression data were used to compare expression across 51 human tissues including liver and LIHC tumor expression. Genes with high expression in Liver and LIHC and low expression across non-liver tissues were considered liver specific and high-quality targets (Figure S3C). This analysis was used as a relative prioritization metric rather than an absolute exclusion threshold and provides a simple insight into the specificity of identified genes for HCC tumor cells. *GPC3*, *ROBO1*, and *MUC13* were confirmed as good tumor liver targets with high LIHC and liver expression whereas *CD24*, *SLC6A8*, and *FXYD3* showed broader off-tissue expression or lower liver/LIHC enrichment. Consequently, *ASPM*, *SLC6A8*, and *FXYD3* were excluded from further validation due to insufficient tissue specificity or lack of reliable antibodies or insufficient tissue specificity, prioritizing markers with higher clinical translatability. Importantly, while *ROBO1* was initially retained as a promising candidate based on these *in silico* liver-specificity matrices, our subsequent multi-level tracking established it as a critical false-positive example from bulk RNA sequencing, highlighting the limitation of relying solely on transcriptomic profiles without empirical protein validation.

### Many *in silico*-identified theranostic markers are abundantly expressed in HCC patient tissue microarrays and associate with clinical outcomes

**Figure 2:**
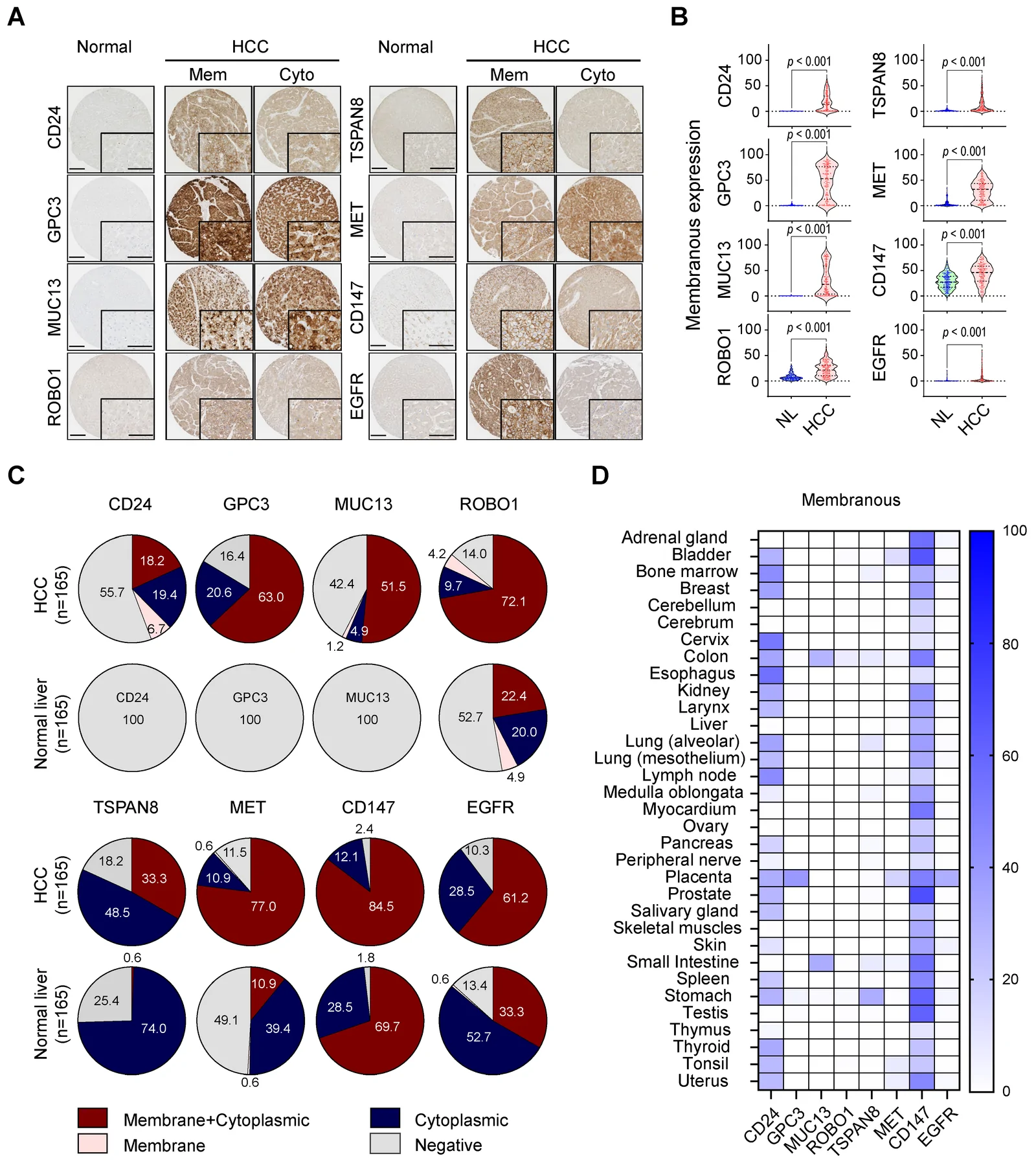
** Profiling of potential theranostic markers in human HCC and normal tissues.** (**A**) Representative immunohistochemical images showing the expression of CD24, GPC3, MUC13, ROBO1, TSPAN8, MET, CD147, and EGFR in formalin-fixed, paraffin-embedded sections of normal and HCC tissues. High magnification images are presented in the inset. Mem, membrane; Cyto, cytoplasm. Scale bar = 200 µm (100 µm in inset). (**B**) Violin plots depicting membranous expression levels of eight potential theranostic markers in 165 HCC patient samples and corresponding non-adjacent normal tissues. Statistical significance was assessed by two-tailed t-test. (**C**) Pie chart illustrates the distribution of expression patterns for the eight theranostic targets, categorized as combined membrane and cytoplasmic, cytoplasmic, membrane, and negative. Data are shown for both HCC tissues (upper panel) and matched non-adjacent normal tissues (lower panel) derived from the 165-patient cohort. Note that CD24, GPC3, and MUC13 show completely negative expression (100% negative, solid gray circles) exclusively in normal liver tissues, contrasting with their elevated expressions in HCC. (**D**) Heatmap comparing protein expression in normal human tissues based on membrane localization (compiled from multiple normal organ TMAs, comprising 33 distinct normal organs and 99 total cores). The heatmap reflects relative protein expression, with darker blue indicating higher levels and lighter blue representing lower levels.

IHC was performed on HCC tissue microarrays to assess the clinical significance of eight candidate therapeutic markers. Seven targets (CD24, GPC3, MUC13, TSPAN8, MET, CD147, and EGFR) were identified through *in silico* analysis, and ROBO1 was included based on a prior literature report 17. Expression levels were evaluated based on their localization to either the membrane or cytoplasm, using digital image analysis (Figure 2A). Among these markers, CD24, GPC3, and MUC13 demonstrated high membrane expression in 24.9% (41/165), 63.0% (104/165), and 52.7% (87/165) of HCC samples, respectively (all *p* < 0.001), but were undetectable in adjacent non-tumor liver tissues (Figure 2B-C). Similarly, TSPAN8, MET, and EGFR were predominantly expressed on the membranes of tumor cells in 33.3% (55/165), 77.6% (128/165), and 61.2% (101/165) of cases, respectively, with minimal presence in non-tumor regions (all *p* < 0.001; Figure 2B-C). ROBO1 and CD147 also showed significantly elevated membrane expression in HCC tissues (both *p* < 0.001), but their expression in non-tumor liver tissues was comparable, suggesting baseline expression of these markers in normal hepatocytes (Figure 2B-C). This specific expression profile formalizes ROBO1 as a definitive false-positive candidate within our screening platform; despite its encouraging RNA-seq enrichment, the persistent baseline expression in normal hepatocytes significantly compromises its tumor-to-normal selectivity at the protein level. Highlighting this distinct mRNA-protein divergence directly underscores the value of our multi-step tissue microarray screening to filter out microenvironmentally unviable targets before downstream radiopharmaceutical formatting.

Next, we examined the correlation between the marker expression and patient survival outcomes. Kaplan-Meier survival analyses demonstrated that patients with high CD24 (*p* = 0.025), MET (*p* = 0.023), and CD147 (*p* = 0.005) membranous expression, as well as those with low MUC13 (*p* < 0.001) membrane expression, had significantly shorter DFS (Figure S4A). Additionally, high TSPAN8 expression correlated with poorer OS (*p* = 0.045; Figure S4B). Cox proportional hazards analyses further confirmed that high MET (HR = 1.727; 95% CI: 1.076–2.773; *p* = 0.024) and high CD147 (HR = 1.869; 95% CI: 1.199–2.912; *p* = 0.006) expression were significantly associated with worse DFS in univariate analysis. Conversely, high MUC13 expression (HR = 0.386; 95% CI: 0.232–0.644; *p* < 0.001) correlated with improved DFS (Table S5). Furthermore, pathologic T-stage (pT) and distant metastasis were identified as independent prognostic factors for DFS, while pT stage alone remained an independent prognostic indicator for OS (Table S5). Cytoplasmic expression was also analyzed and is summarized in Figure S5 and S6. Importantly, while expression-survival correlations can be hypothesis generating, further validation is needed.

In the selection of tumor-enriched targets for therapeutic development, it is critical to assess protein levels in normal tissue as well to rule in or out potential (e.g. on target, off tumor) toxicity. Accordingly, we assessed their presence across various normal human tissues using normal tissue microarrays. Immunohistochemical analysis quantified both membranous and cytoplasmic expression. Membranous GPC3 and EGFR were predominantly expressed in the placenta, with minimal detection in other normal organs (Figure 2D). Notably, MUC13 and TSPAN8 showed peak expression in the colon and small intestine, respectively, while ROBO1 had negligible expression across all tested normal organs. Moderate MET expression was observed in the placenta and bladder. Meanwhile, CD24 and CD147 displayed moderate to strong membranous expression across multiple tissues. In sum, our normal tissue TMA assessment suggests that GPC3, MUC13, ROBO1, TSPAN8, MET and EGFR appear to exhibit low normal tissue expression at the protein level, suggesting that these represent promising theranostic candidate targets for HCC. However, CD24 and CD147 may be less attractive for such purposes, unless alternate modes of administration (e.g., transarterial) are considered to avoid off-tumor binding and subsequent toxicity.

### Combinations of markers could begin to address target expression heterogeneity in HCC

**Figure 3:**
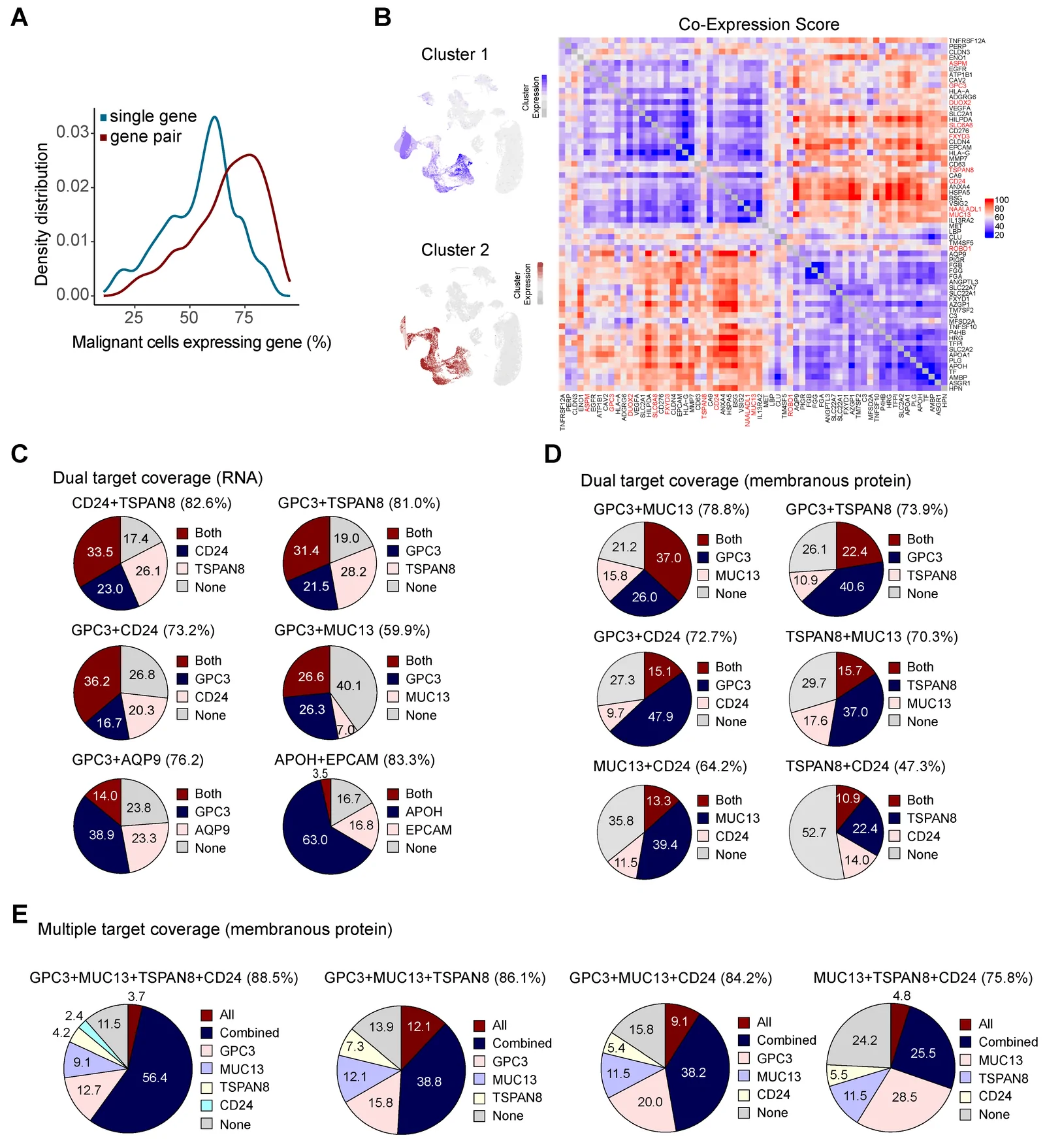
** Distribution and coverage of dual and multiple target combinations in HCC based on membrane expression of GPC3, MUC13, TSPAN8, and CD24.** (**A**) Distribution of malignant-cell coverage for differentially expressed malignant-cell-enriched membrane-associated genes (n = 63). The distribution shows the percentage of malignant cells expressing each individual gene (blue) or at least one gene in each possible gene pair (red). All pairwise combinations among the 63 malignant-cell-enriched genes were evaluated. The median malignant-cell coverage was approximately 60% for individual genes and approximately 75% for gene pairs. (**B**) Heatmap of paired Co-Expression scores between differentially expressed malignant-cell-enriched genes. Genes are separated into 2 clusters identified by a weighted gene co-expression network analysis (WGCNA). The average gene expression for genes in Cluster 1 (blue) and Cluster2 (maroon) are projected onto a UMAP of all cells (left). The 10 genes identified as differentially expressed in both the single cell (malignant vs. non-malignant) and RNA-seq (tumor vs. normal) datasets are highlighted in red text. (**C**) Distribution of dual target combinations among select differentially expressed malignant enriched genes based on percentage of malignant cells expressing the gene. The sections of the pie chart indicate the percentage of cells expressing both genes (Maroon), individual genes (blue and pink) or neither gene (grey). (**D**) Distribution of dual target combinations among GPC3, MUC13, TSPAN8, and CD24 in HCC patients based on membrane expression. The pie chart displays the percentage coverage across 165 HCC patients, categorized into both targets, single target, and none. (**E**) Distribution of multiple target combinations among GPC3, MUC13, TSPAN8, and CD24 in HCC patients based on membrane expression. The pie chart illustrates the percentage coverage in the same cohort (n = 165), categorized into all targets, combinations of two or more targets, single target, and none.

Given the heterogeneity of HCC, identifying novel combinatorial of the eight candidate markers presents a strategy to identify and treat a greater proportion of HCC tumors. We hypothesized that a subset of gene pairs expressed in high percentage of malignant cells could serve as a more effective, general HCC signature. We determined that the majority of differentially expressed genes were expressed in ~60% of malignant cells (Figure 3A, blue line) while *AMBP* was expressed in the highest percentage of malignant cells, at 84.4%. We then calculated the percentage malignant cells expressing either gene for all possible pairs of single-cell differentially expressed genes and observed a marked increase relative to individual genes alone (Figure 3A red line). When gene pairs were considered, we saw that ~75% of malignant cells were targeted for most gene pairs.

Using WGCNA on the malignant, single-cell differentially expressed genes, we identified two clusters of highly correlated gene expression (Figure 3B). The average gene expression in each cluster shows the specificity for malignant cells. A Co-expression score was calculated (see methods) to identify which gene pairs provide the greatest improvement in targeting malignant cells as a percentage compared to an individual gene. Gene pairs drawn from the same cluster produced the lowest scores, consistent with expression in overlapping malignant cells. By contrast gene pairs spanning different clusters produced the highest scores, indicating that they captured complementary malignant cell subsets and maximized the increase in malignant cells captured over either gene alone (Figure 3B). Notably, genes with high co-expression scores did not necessarily target a high percentage of malignant cells individually; however, when gene pairs were considered together, the proportion of targeted malignant cells increased substantially (Figure 3C). For example, *CD24*+*TSPAN8* (both Cluster1) and *APOH*+*EPCAM*, (Cluster 1 + 2) captured a similar overall percentage of malignant cells, approximately 83%, but resulted in very different co-expression scores. *CD24* was expressed in 56.5% of malignant cells, *TSPAN8* was expressed in 59.6%, and 33.5% of malignant cells co-expressed both markers, resulting in a co-expression score of 26.1. By contrast, *APOH* was expressed in 66.5% of malignant cells, *EPCAM* was expressed in 20.3%, and only 3.5% of malignant cells co-expressed both markers, resulting in a higher co-expression score of 63.0. Thus, although both marker pairs captured a similar percentage of malignant cells overall, *APOH*+*EPCAM* represents a more complementary gene pair that identifies more distinct malignant-cell populations. Although *AMBP* emerged as the most robust single-gene candidate with 84.4% coverage. in our combinational analysis, further in-depth validation of *AMBP* on clinical samples were temporarily deferred due to the current unavailability of validated commercial antibodies. Therefore, to optimize clinical translation and maintain experimental feasibility, we focused our downstream validation on high-scoring candidates within these clusters that possessed both high liver-specificity and reliable, established detection reagents.

All genes shared between the single cell and TCGA differential genes, with the exception of *ROBO1*, localized to Cluster 1. Individually these genes were expressed in only ~10-50% of malignant cells (Figure S3B). However, when paired with genes from Cluster 2, their coverage of malignant cells increased substantially. The gene pair score can help identify potential targets that might otherwise have been overlooked based on their limited expression in malignant cells, but become informative when paired with complementary markers. As our primary goal was to identify the most translatable theranostic candidates, we focused our validation on markers within these clusters that demonstrated high liver-specificity and the availability of reliable detection reagents.

In addition to evaluating multi-target combinations at the transcriptome level, we validated marker co-expression at the protein level using IHC analysis. For dual marker combinations based on membrane expression, the combination of GPC3 and MUC13 showed the highest coverage rate of 78.8% (129/165), followed by the combinations of GPC3 and TSPAN8 at 73.9% (122/165), GPC3 and CD24 at 72.7% (120/165), TSPAN8 and MUC13 at 70.3% (116/165), MUC13 and CD24 at 64.2% (106/165), and TSPAN8 and CD24 at 47.3% (78/165) (Figure 3D). Among the multiple marker combinations, the combination of GPC3+MUC13+TSPAN8+CD24 achieved the highest patient coverage rate of 88.5% (146/165). Three-marker IHC combinations demonstrated the following coverage rates: GPC3+MUC13+TSPAN8 at 86.1% (142/165), GPC3+MUC13+CD24 at 84.2% (139/165), and MUC13+TSPAN8+CD24 at 75.8% (125/165) (Figure 3E). Notably, theranostic marker expression in HCC patients exhibits marked heterogeneity (Figure S7).

Together, these findings underscore the potential of using a combinational marker strategy to overcome the challenge of target expression heterogeneity of target expression in the development of HCC-selective diagnostic and therapeutic agents. While no single agent that is specific to two or more targets are currently approved, several are under investigation, and it is not outside the realm of possibility to engineer such multimers composed of low molecular weight biomolecules, such as small molecules, peptides, or mini-proteins, for example.

### Potential tumor-enriched markers are present in various liver cancer cell lines

**Figure 4:**
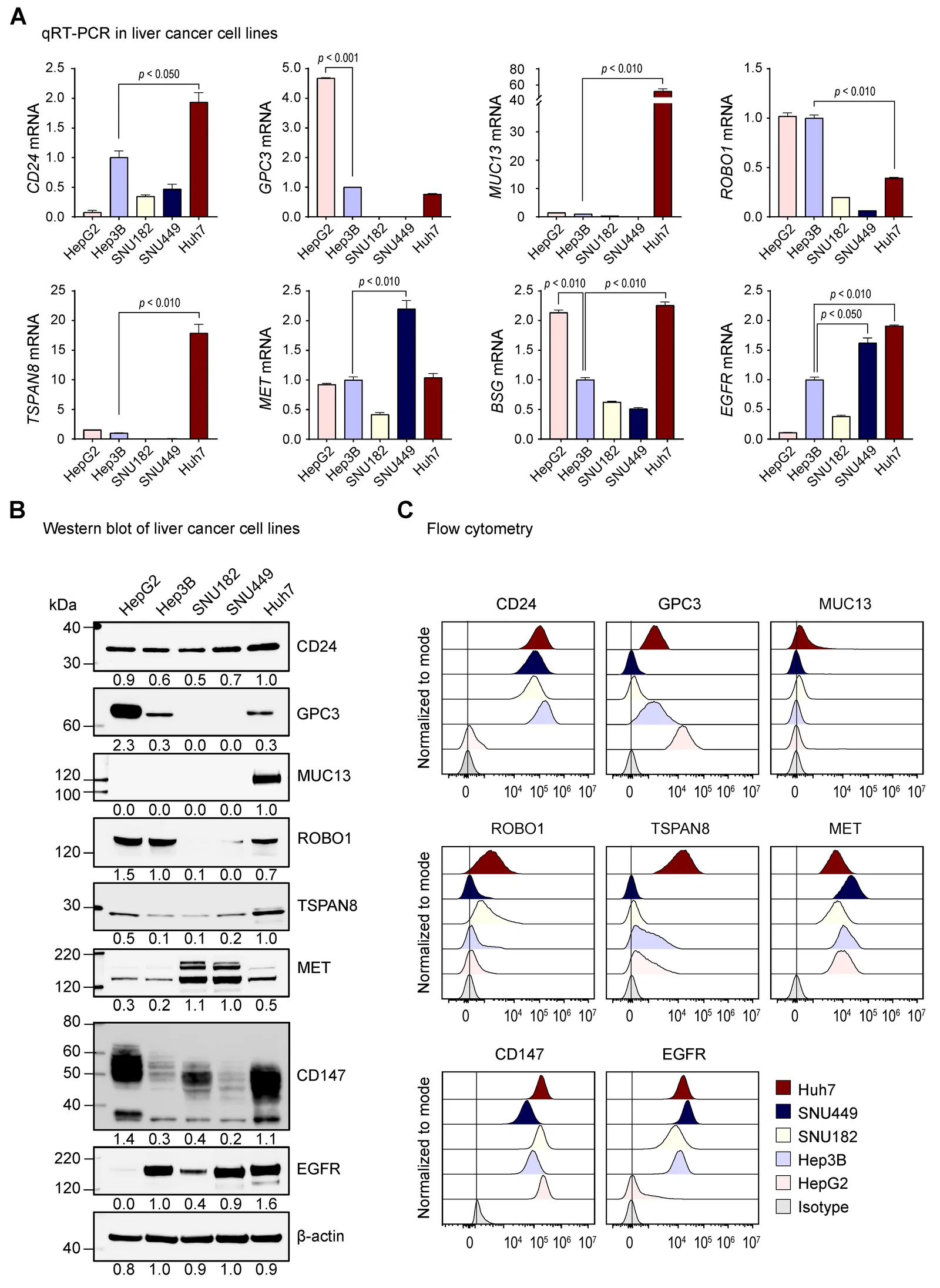
** Validation of theranostic targets in liver cancer cell lines.** (**A**) Quantitative real-time RT-PCR analysis of the gene expression levels of *CD24*, *GPC3*, *MUC13*, *ROBO1*, *TSPAN8*, *MET*, *BSG* (CD147), and *EGFR* in liver cancer cell lines. *ACTB* served as an internal control, and relative expression levels were normalized to Hep3B. Data represent the mean ± standard deviation (SD) of three independent experimental replicates (n = 3). Statistical significance was assessed by two-tailed t-test. (**B**) Western blot analysis of the identified theranostic targets in liver cancer cell lines, with β-actin as the internal control. The numbers beneath the blot images represent the expression levels, shown as fold-change relative to the control. (**C**) Flow cytometry analysis of membranous expression of eight potential theranostic targets across five liver cancer cell lines, with stacked histograms reflecting independent cellular distribution measurements. Solid lines represent cells labeled with the isotype control.

To rigorously validate whether the HCC-enriched molecules were valuable as theranostics targets, we first needed to assess their expression in commercially available cell lines. We performed quantitative real-time PCR (qRT-PCR) to assess mRNA expression levels of eight theranostic markers in five liver cancer cell lines: HepG2, Hep3B, SNU182, SNU449, and Huh7 (Figure 4A).

Among the markers analyzed, *CD24* exhibited the highest expression in Huh7 cells, showing a 1.9-fold (*p* < 0.050) higher expression compared to Hep3B. Similarly, *MUC13* and *TSPAN8* were predominantly upregulated in Huh7, displaying 52.3-fold (*p* < 0.010) and 17.9-fold (*p* < 0.010) higher expression levels, respectively, relative to Hep3B. *GPC3* was significantly upregulated in HepG2 cells (4.7-fold higher than Hep3B, *p* < 0.001), whereas SNU182 and SNU449 exhibited minimal or undetectable levels. *ROBO1* expression was comparable between HepG2 and Hep3B, whereas *EGFR* levels were similarly increased in SNU449 and Huh7, with 1.6-fold (*p* < 0.050) and 1.9-fold (*p* < 0.010) elevations, respectively, compared to Hep3B. *MET* expression varied across cell lines, with SNU449 exhibiting the highest expression (2.2-fold increase, *p* < 0.010), while HepG2 and Huh7 displayed comparable upregulation of *BSG*, with 2.1-fold (*p* < 0.010) and 2.3-fold (*p* < 0.010) increases, respectively, over Hep3B.

To confirm these findings at the protein level, we performed western blot analysis (Figure 4B), which corroborated the qRT-PCR results. Specifically, GPC3, MUC13, and TSPAN8 exhibited strong expression in HepG2 (2.3-fold), and Huh7 (1.0-fold), respectively, compared to Hep3B. The expression patterns of CD24, MET, and CD147 varied among the liver cancer cell lines. ROBO1 was primarily detected in HepG2, Hep3B, and Huh7, while its expression was negligible in SNU182 and SNU449. EGFR was widely expressed in four HCC cell lines (Hep3B, SNU182, SNU449, and Huh7).

Given the importance of membrane-bound markers for radiotheranostic applications, we further assessed their surface expression. Flow cytometry assays showed high expression of CD24 and EGFR in four HCC cell lines, whereas expression in HepG2 was minimal (Figure 4C). The surface expression patterns of GPC3 aligned with qRT-PCR and Western blot data, showing high levels in HepG2, Hep3B, and Huh7. Additionally, MET and CD147 were consistently expressed on the surface of all five liver cancer cell lines. TSPAN8 was prominently detected on the membrane of Huh7 cells, consistent with its transcriptional expression. Meanwhile, MUC13 demonstrated low surface expression across all tested liver cancer cell lines. These findings show that our HCC-selective markers exhibit distinct expression profiles across liver cancer cell lines, confirming the heterogeneity in liver cancer oncogenesis and the need for rigorous characterization to develop diverse targeted therapeutic interventions.

### Target-selective immunoPET agents successfully localize to liver cancer xenograft tumors

**Figure 5:**
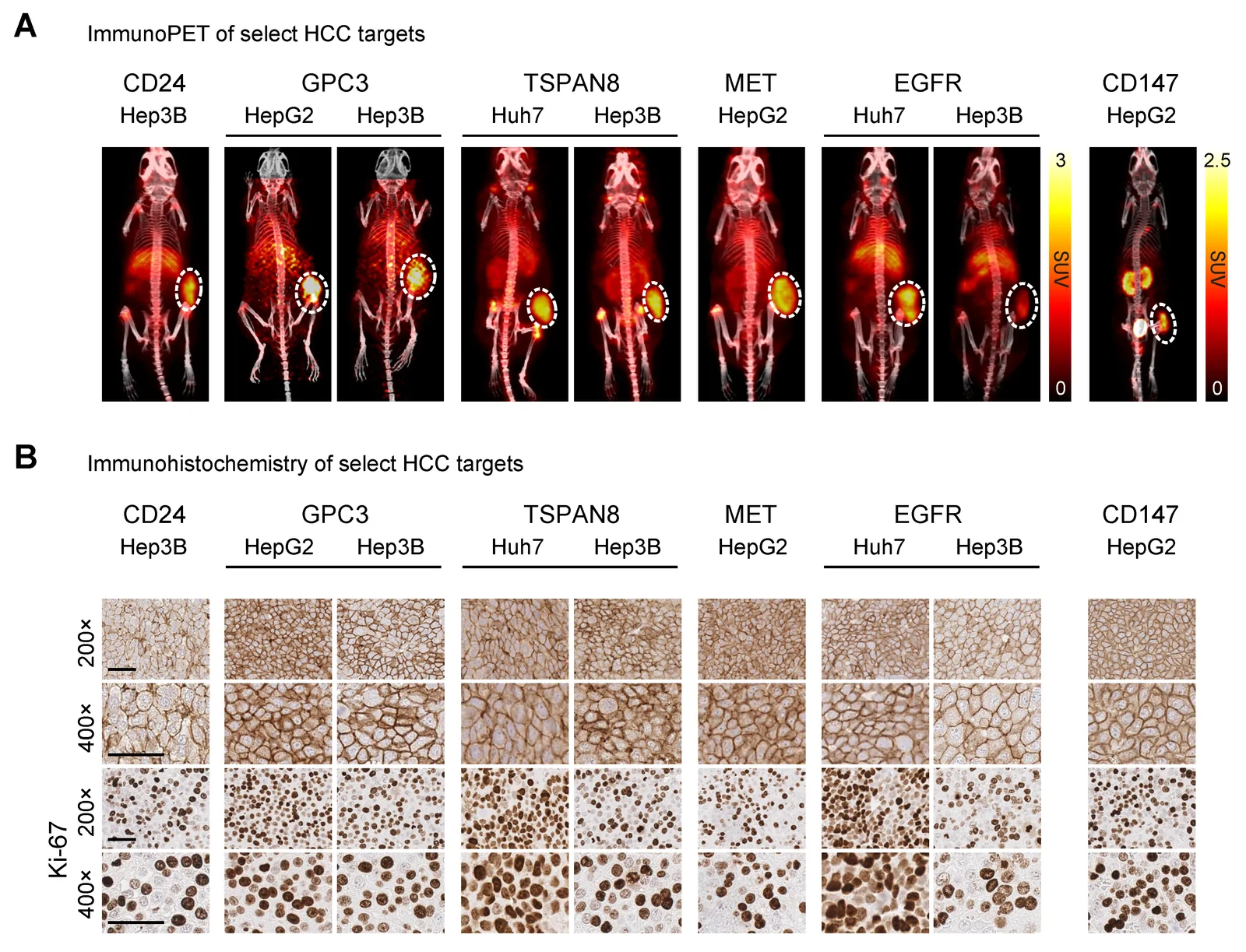
** Confirmation of theranostic marker accumulation and expression using ImmunoPET and immunohistochemistry.** (**A**) Validation of targeted theranostic marker detection via immunoPET in xenograft models. Representative MIP-PET/CT images of mice bearing liver cancer cell line xenografts, injected with ^89^Zr-DFO-labeled antibodies (anti-CD24, anti-GPC3, anti-TSPAN8, anti-MET, and anti-EGFR) or [^18^F]FPy-DMN1 (for CD147 targeting), and imaged at target-specific time points (2, 72, or 144 h) post-injection. Tumors are highlighted by white dotted circles. PET images are presented with radioactivity levels calibrated in standardized uptake values (SUV). Quantitatively, the SUV_mean_ for the TSPAN8 panel at 144 h is 0.21 ± 0.05, whereas the SUV_mean_ for the GPC3 panel at 72 h is 0.85 ± 0.18. Representative qualitative panels are systematically compiled and cross-referenced from independent, peer-reviewed source publications 42, 60, 64 to serve as foundational visual confirmation of downstream surface accessibility. (**B**) Representative immunohistochemical images of xenograft tissue samples showing the expression of CD24, GPC3, TSPAN8, MET, EGFR, CD147, and Ki-67 following immunoPET. Images are provided at both low (top panels) and high (bottom panels) magnifications. Scale bar = 50 µm (indicated in the leftmost panels and applicable to all panels in the respective rows).

To evaluate the potential of the identified markers in this study as theranostic targets for liver cancer, we administered the ^89^Zr- or ^18^F-labeled antibody conjugates and performed immunoPET imaging in several murine models of liver cancer using various cell lines. The imaging results demonstrated excellent tumor-to-background with significant accumulation of the radiolabeled probes at the tumor sites (Figure 5A). Generally, off-target signals were minimal, confirming the utility of diagnostic imaging of the evaluable targets. Notably, while the SUV_mean_ for the TSPAN8 panel at 144 h appeared modest (0.21 ± 0.05), reflecting its baseline expression density and extended clearance, it remained evaluable in comparison with the robust signal of the GPC3 panel at 72 h (0.85 ± 0.18).

In parallel, marker expression in the liver cancer xenografts was validated via IHC analysis (Figure 5B**)**. Staining results confirmed high membrane expression of the evaluated markers within tumor tissues. Furthermore, a significant positive correlation was observed between target expression levels quantified by IHC and both SUV_mean_ (*Pearson’s ρ* = 0.707, *p* = 0.033) and SUV_max_ (*Pearson’s ρ* = 0.723, *p* = 0.028) and target expression levels quantified by IHC (Figure S8). Additionally, Ki-67 expression showed similar levels across xenograft tissues derived from liver cancer cells. These promising immunoPET results highlight the potential of these markers to serve as both diagnostic biomarkers and as candidates for targeted therapy, specifically, and highlight the utility of our systematic approach for identifying and validating putative targets more generally. Such work is critical in establishing proof-of-concept for the targets prior to launching more rigorous biomolecule development and optimization.

## Discussion

Here, we identify several HCC-enriched molecules and demonstrate their potential to guide the development of novel diagnostic and therapeutic agents. By utilizing publicly available bulk and single-cell RNA datasets alongside validation through patient-derived tissue microarrays and liver cancer cell lines, we established a robust framework for target discovery. Our approach builds upon prior efforts to identify the diagnostic foundations of radiopharmaceutical targets 48 with experimental validation studies. These efforts may facilitate the repurposing of existing targeted therapies approved for other indications or the development of entirely new agents for HCC patients and beyond.

Some of the targets we identified have been reported in the literature, however, at the time of this writing, only GPC3- and CD147-targeted agents have been evaluated in HCC clinical trials 18, 21, 49, 50. Importantly, the GPC3-targeted antibody, codrituzumab, has previously been studied in a phase 2 clinical trial of patients with advanced HCC randomized to either codrituzumab or placebo 18. This trial was negative for its primary endpoint of progression-free survival and secondary endpoint of overall survival. These data suggest that antibody-dependent cellular cytotoxicity, the putative mechanism of action for unconjugated antibodies, is insufficient to exert a therapeutic benefit despite the codrituzumab having a very high affinity for GPC3 and demonstrating good HCC localization in humans 22. For such targets, it is not unreasonable to consider coupling a cytotoxic cargo to the targeting biomolecule (e.g., antibody, peptide) and enhancing cytotoxicity for therapy. In the radiopharmaceutical realm, the cargo could be either a radionuclide with diagnostic or therapeutic emissions 24-27, 51-53. To date, there are two GPC3-specific radiopharmaceutical therapy agents, one based on a cyclic peptide (NCT06726161) and another based on a full-length antibody (NCT06764316) are being tested in prospective clinical trials using the therapeutic alpha particle emitter actinium-225.

The clinical viability of CD147 as a therapeutic target in HCC is evinced by the success of ^131^I -metuximab, a CD147-specific F(ab’)_2_ fragment approved in China. In a phase 2 randomized controlled clinical trial, this treatment was shown to improve 5-year relapse-free survival compared to placebo in patients with HCC following surgical resection 21. This work underscores the potential of CD147-targeted radiopharmaceutical agents in the treatment of HCC, specifically, and perhaps, the modality’s efficacy more generally. Importantly, this agent is administered transarterially, suggesting that this approach may allow for therapy despite the significant non-tumor tissue expression of CD147. Building on this therapeutic foundation, we recently developed and validated [^18^F]FPy-DMN1, a novel CD147-targeted nanobody PET tracer. This agent exhibits high binding affinity (*K_D_* = 0.71 ± 0.1 nM) and excellent tumor-to-background ratios in murine models 42. Crucially, our quantitative *in vivo* evaluations demonstrated that despite moderate static baseline expression of CD147 in certain normal compartments, [^18^F]FPy-DMN1 achieved rapid blood clearance and high contrast within 2 h post-injection, yielding a robust tumor accumulation of 3.82 ± 0.93 %IA/g, a tumor-to-blood ratio of 18.7 ± 3.9, and a tumor-to-liver ratio of 3.69 ± 0.67, while effectively minimizing kidney background to a 0.83 ± 0.24 tumor-to-kidney ratio. In addition to GPC3 and CD147, we identified several other putative tumor-enriched targets with theranostic potential including CD24, TSPAN8, MET, MUC13, and EGFR. While some had been previously described, many were understudied in HCC, especially with respect to radiotheranostic applications.

CD24 is a glycoprotein frequently overexpressed in cancers such as breast, lung, hepatocellular, and ovarian cancers, where it promotes tumor progression, metastasis, and poor prognosis. It drives oncogenesis by regulating key signaling pathways, including Src/STAT3, WNT/β-catenin, and EGFR, and contributes to drug resistance, such as sorafenib resistance in HCC 54. While CD24 has been pursued as therapeutic target for ADCs 55-57 and cell therapies 58, 59, these approaches often encounter limitations such as off-target toxicities in normal tissue or challenges with sustained clinical efficacy in solid tumors. Our group was first to evaluate its potential as a candidate for radiotheranostic translation and reported a more detailed characterization in a separate manuscript 60. In our detailed evaluation of the full-length CD24-targeted tracer [^89^Zr]Zr-DFO-α-hCD24, *ex vivo* biodistribution and PET quantifications in Hep3B xenografts confirmed a distinct tumor accumulation of 8.7 ± 3.2 %IA/g and an SUV_max_ of 2.7 ± 0.8 at 144 h post-injection, establishing a tumor-to-liver ratio of 0.8 ± 0.1 along with expected clearance profiles in the spleen (19.4 ± 2.3 %IA/g) and liver (11.8 ± 0.8 %IA/g). When compared directly with the aforementioned CD147 nanobody metrics, these highly divergent *in vivo* distribution patterns provide essential insights within our broader screening platform of eight tested candidates. While the full-length IgG molecule exhibits predictable off-target retention within the mononuclear phagocyte system, the small nanobody constructs shifts the primary off-target background to the renal excretion pathway. These well-described biodistribution differences based on biomolecule platform can be used to tune on-target/off-tumor toxicity during subsequent therapeutic translation.

The tetraspanins are glycoproteins involved in key cellular processes such as migration and growth. Tetraspanin-8 (TSPAN8) is overexpressed in several cancers, including ovarian 61, HCC, gastric and colorectal cancer (CRC) 62 and is associated with poor patient prognosis. While a prior study demonstrated that their full-length antibody-based radiotheranostic agents ([^111^In]In-Ts29 and [^177^Lu]Lu-Ts29) could specifically localize to and treat preclinical models of CRC 63, our recent work has established TSPAN8 as a novel, tumor-selective immunoPET target for HCC 64. This study shows TSPAN8 as a compelling alternative for the subset of GPC3-low HCC cases, successfully establishing it as viable target for next-generation radiopharmaceutical development.

MET, also known as the hepatocyte growth factor receptor (HGFR), is a receptor tyrosine kinase that plays a crucial role in various cellular processes 65. Dysregulation of this pathway, including overexpression or activating mutations of MET, is commonly associated with tumorigenesis and metastasis in various cancers, including HCC 66, 67. Several imaging agents targeting MET are being developed or studied for MET aberrant cancers utilizing MET overexpression to enhance tumor detection and characterization, however, most studies have focused on non-HCC histologies 43, 68, 69. Our prior work successfully testing the MET-specific antibody onartuzumab as a targeting vector in models of pancreatic adenocarcinoma as a radiotheranostic agent in that disease, prompted us to assess its utility in HCC after identifying MET as a potential target for the disease. The results presented here highlight the target’s utility as an imaging biomarker to improve diagnostic precision. Furthermore, the high prevalence of MET overexpression in HCC also provides a compelling rationale for its use in future radiotheranostic applications. In this framework, MET-targeted imaging could serve as a prerequisite for identifying patients most likely to benefit from MET-directed radiopharmaceutical therapies or other targeted interventions.

MUC13, another HCC-selective marker we identified, is a high-molecular-weight transmembrane glycoprotein that is frequently overexpressed in various cancers, including HCC 70. Its elevated expression in HCC has been linked to tumor progression and an increased hepatitis B virus copy number 71. Furthermore, MUC13 contributes to the activation of the Wnt/β-catenin signaling pathway by facilitating the nuclear translocation of β-catenin, which upregulates downstream effectors like c-Myc and cyclin D 72. A recent abstract reported on successful visualization of MUC13 by PET/CT using a ^89^Zr-labeled full length antibody in a xenograft mouse model of CRC 73. In our study, we observed that MUC13 expression was higher in HCC compared to normal liver tissue, with minimal cross-reactivity in other normal organs, save for colon and small intestine. These results highlight the potential of MUC13-targeted immunoPET for future radiotheranostic applications in HCC. Unfortunately, at the time of this writing, we were unable to secure a good biomolecule specific to MUC13 to perform immunoPET studies. To overcome this technical bottleneck, evaluating custom-engineered MUC13-specific targeting vectors via alternative selection platforms is underway as an independent follow-up study.

EGFR is a well-established receptor tyrosine kinase that has been a target for several malignancies, and HCC is no exception. In fact, a phase 3 clinical trial evaluating, the then standard-of-care sorafenib, a pan-tyrosine kinase inhibitor, alone versus sorafenib in combination with erlotinib, a small molecule tyrosine kinase inhibitor (TKI) of EGFR, was reported in 2015 74. The results showed that erlotinib did not improve the primary endpoint of survival. Still, given the overexpression of EGFR in HCC, Takao et al. leveraged this feature to successfully deliver EGFR-targeted photodynamic immunotherapy in preclinical models of HCC 75. Work evaluating EGFR as a radiopharmaceutical therapy in preclinical models of HCC is currently under review.

Curiously, while ROBO1, the human homolog for the *Drosophila* roundabout gene, has been evaluated for radiotheranostic potential 17 and our RNA analyses suggested it could be promising, we concluded it to be less compelling for future development. This prioritization shift was based on a lack of robust protein-level support; specifically, we observed relatively low expression in tumor TMAs and commercially available cell lines. Although it is possible that ROBO1 is expressed in a high proportion of HCC at very low levels, our workflow required high-density surface expression to identify a viable target. Consequently, ROBO1 did not meet the criteria established for a compelling immunoPET or therapeutic candidate in this study. This prominent mRNA-protein discordance highlights a major logistical strength of our multimodal pipeline, positioning ROBO1 as a valuable case study demonstrating that rigorous experimental protein validation is entirely indispensable to prevent the premature pursuit of targets that lack sufficient surface density. Biologically, these discrepancies are often governed by complex translational efficiency and regulated protein degradation pathways that do not align with transcript abundance, while technically, they underscore that broad in silico transcriptomic screening cannot substitute for empirical tissue-level validation due to variations in antibody affinity and epitope accessibility. Notably, the previous publication showed poor tumor-background ratio in preclinical biodistribution studies, confirming our conclusion.

We note that the multi-target combination models presented herein represent a predictive, data-driven framework based on histopathological co-expression, rather than an immediate experimental evaluation of multi-specific therapeutic constructs. While testing simultaneous combination imaging using the unoptimized single target tool agents developed here would not be technically sound, the clinical translation and regulatory approval of multi-specific platforms, such as the EGFR-MET bispecific antibody amivantamab, strongly support the conceptual merit of targeting co-expressed candidate networks. However, the realistic clinical implementation of such multi-target strategies requires careful consideration of translational logistics. Administering a cocktail of separate radiopharmaceutical probes introduces notable clinical complexities, including fragmented diagnostic imaging workflows, cumulative radiation dose summation concerns, and intricate regulatory approval hurdles for multi-component regimens. To circumvent these translational barriers, future directions could prioritize the engineering of single-molecule bispecific or multispecific theranostic agents. Leveraging low-molecular-weight biomolecular platforms, such as multivalent peptides, mini-proteins, or nanobody fusions, offers a viable strategy to simultaneously engage complementary surface antigens within a single, streamlined dosing framework, effectively addressing tumor heterogeneity while maintaining clinical and regulatory feasibility. We present these high-yield target combinations as a foundational blueprint to guide subsequent bioengineering efforts, wherein the design, formulation, and therapeutic validation of novel multi-specific binders remain an active, independent focus of future investigation. Within this translational framework, we acknowledge as a limitation of this study that our current *in vivo* validation is restricted to diagnostic immuno-PET imaging, and we have not yet evaluated therapeutic radionuclides (e.g., ^177^Lu or ^225^Ac) or conducted corresponding efficacy and toxicity studies. Furthermore, it is critical to recognize that the human cell line-derived subcutaneous mouse xenograft models employed in this screening study cannot fully recapitulate the complex, multi-layered tumor microenvironment or the exact clinical target expression levels found in human HCC patients. Particularly for microenvironment-sensitive targets such as MET and EGFR, their clinical expression levels can be dynamically induced or altered by physiological stressors within the clinical tumor niche, such as hypoxia, which may not be fully recapitulated in standard preclinical models. In the paradigm of radiotheranostic development, however, confirming robust *in vivo* target engagement through highly selective imaging is a mandatory prerequisite. While this work successfully establishes that foundational diagnostic arm, future studies which are currently ongoing are warranted to pair these prioritized targeting vectors, comprising both the optimized single-agent platforms and prospective multi-specific constructs, with therapeutic radioisotopes to achieve a complete radiotheranostic validation.

Beyond the tumor-enriched targets, we also interrogated the possibility of tumor microenvironment targets, including *FOLH1* (a.k.a. PSMA), which is expressed in the neovasculature of some tumors including HCC as well as FAP, which is expressed on fibroblasts, for which there are available radiotheranostic agents that are either approved or being tested clinically. Because tumor-selective imaging would be beneficial in diagnosis, surveillance, and treatment responses monitoring for patients with HCC, both targets are actively being evaluated in clinical studies in patients with HCC 13, 76-79.

We should emphasize that the primary purpose of our efforts was to identify and validate the putative HCC targets for diagnostic and therapeutic development, not to present the immunoPET agents generated as candidates for further development themselves. For example, having demonstrated that some of these markers have high expression in normal tissue, one could either decline pursuit of that target or attempt to balance tumor targeting accessibility and on-target, off tumor potential for toxicity. Thus, the framework presented here can help distinguish targets to be further explored from those that should not.

In conclusion, we have identified and validated several highly targeted and tumor-enriched HCC targets that can be leveraged for novel diagnostic and therapeutic agents. By identifying targets overrepresented on the plasma membrane, we provide a blueprint for managing transcript-protein discordance in target discovery. The ability to mix and match multiple targets may help address tumor heterogeneity and maximize treatment efficacy. While this data-driven framework remains highly promising for HCC target prioritization, its generalized implementation across other malignancies may face significant logistical constraints in tumor types where high-quality single-cell RNA sequencing data or comprehensive tissue microarray resources are currently scarce or unavailable. Nonetheless, this programmatic workflow offers a structured, preliminary blueprint for target discovery and prioritization that could be extended to other cancer histologies to systematically expand the hypothesis-generating landscape of targeted radiopharmaceuticals.

## Supplementary Material

Supplementary results, figures and tables 2-4.

Supplementary table 1: UniProt cell membrane R.

## Abbreviations

HCC: Hepatocellular Carcinoma
TMAs: Tissue Microarrays
ImmunoPET: Immuno-Positron Emission Tomography
ADC: Antibody-Drug Conjugates
SSTR: Somatostatin Receptor
PSMA: Prostate Specific Membrane Antigen
GPC3: Glypican-3
BSG: Basigin
TCGA: The Cancer Genome Atlas
LIHC: Liver Hepatocellular Carcinoma
NIH: National Institutes of Health
NIDAP: NIH Integrated Data Analysis Portal
nTPM: normalized Transcripts Per Million
pTPM: protein-coding Transcripts Per Million
CPM: Counts Per Million
CCA: Canonical Correlation Analysis
PCA: Principal Component Analysis
ATCC: American Type Culture Collection
DMEM: Dulbecco’s Modified Eagle’s Medium
RPMI: Roswell Park Memorial Institute
EMEM: Eagle’s Minimal Essential Medium
IHC: Immunohistochemistry
SDS-PAGE: Sodium Dodecyl Sulphate-Polyacrylamide Gel Electrophoresis
CNVs: copy-number variations
PADB: Protein Atlas Database
WGCNA: Weighted Gene Co-expression Network Analysis
qRT-PCR: quantitative real-time PCR
TSPAN8: Tetraspanin-8
CRC: Colorectal Cancer
HGFR: Hepatocyte Growth Factor Receptor
TKI: Tyrosine Kinase Inhibitor
