# Supplementary material for: A multimodal pipeline for the identification and diagnostic immunoPET validation of hepatocellular carcinoma targets for radiotheranostic development
Source: Theranostics. 2026 Jul 29;16(15):8501–20. doi: 10.7150/thno.136906 (PMC13549132; doi:10.7150/thno.136906)
Supplement: Supplementary file 1 [file thnov16p8501s1.pdf]

[Supplementary Materials]

Supplementary Results

Cytoplasmic expression of *in silico*-identified theranostic markers are highly expressed in HCC patient samples and associated with clinical outcomes

Cytoplasmic expression of these markers, CD24, GPC3, MUC13, ROBO1, MET, and CD147 were significantly overexpressed in HCC compared to non-tumor tissues (all $p < 0.001$), while TSPAN8 displayed a moderate increase ($p < 0.050$). EGFR cytoplasmic expression did not show significant differences between HCC and non-tumorous tissues (Figure S5A).

In the analysis of cytoplasmic expression of selected markers in normal tissue microarrays, a comparable pattern of localization was observed. The markers CD24, GPC3, MUC13, ROBO1, TSPAN8, MET, and CD147 demonstrated cytoplasmic distribution patterns that closely mirrored their membranous expression. However, EGFR showed a notably higher cytoplasmic expression in several normal tissue types (Figure S5B). Survival analysis revealed that high cytoplasmic expression of CD24 ($p = 0.003$), ROBO1 ($p = 0.024$), and CD147 ($p = 0.001$) was significantly associated with shorter DFS (Figure S5C). In addition, low MUC13 cytoplasmic expression correlated with poorer DFS ($p = 0.002$), while increased MET cytoplasmic expression was linked to both worse DFS ($p = 0.002$) and OS ($p = 0.029$) (Figure S5C-D). Multivariate Cox analysis identified high ROBO1 cytoplasmic expression as a significant prognostic predictor for DFS (HR = 2.519; 95% CI: 1.468–4.322; $p = 0.001$). Furthermore, high CD147 cytoplasmic expression was found to be an independent predictor for both DFS (HR = 1.866; 95% CI: 1.067–3.264; $p = 0.029$) and OS (HR = 4.250; 95% CI: 1.397–12.925; $p = 0.011$). High CD24 cytoplasmic expression was also identified as an independent prognostic factor for DFS (HR = 1.799; 95% CI: 1.023–3.164; $p = 0.041$) (Table S5).

For combinations based on cytoplasmic expression, dual marker combinations showed HCC coverage rates ranging from 74.6% for MUC13+CD24 to 97.0% for GPC3+TSPAN8 (Figure S6A). Additionally, the combination of multiple markers based on cytoplasmic expressions resulted in HCC patient coverage rates ranging from 93.9% to 98.8% (Figure S6B). Similarly, the combination of dual and multiple markers that incorporate both membranous and cytoplasmic expressions showed comparable HCC patient coverage, with the highest coverage rates of 97.0% for dual marker combinations (Figure S6C) and 98.8% for multiple marker combinations (Figure S6D).

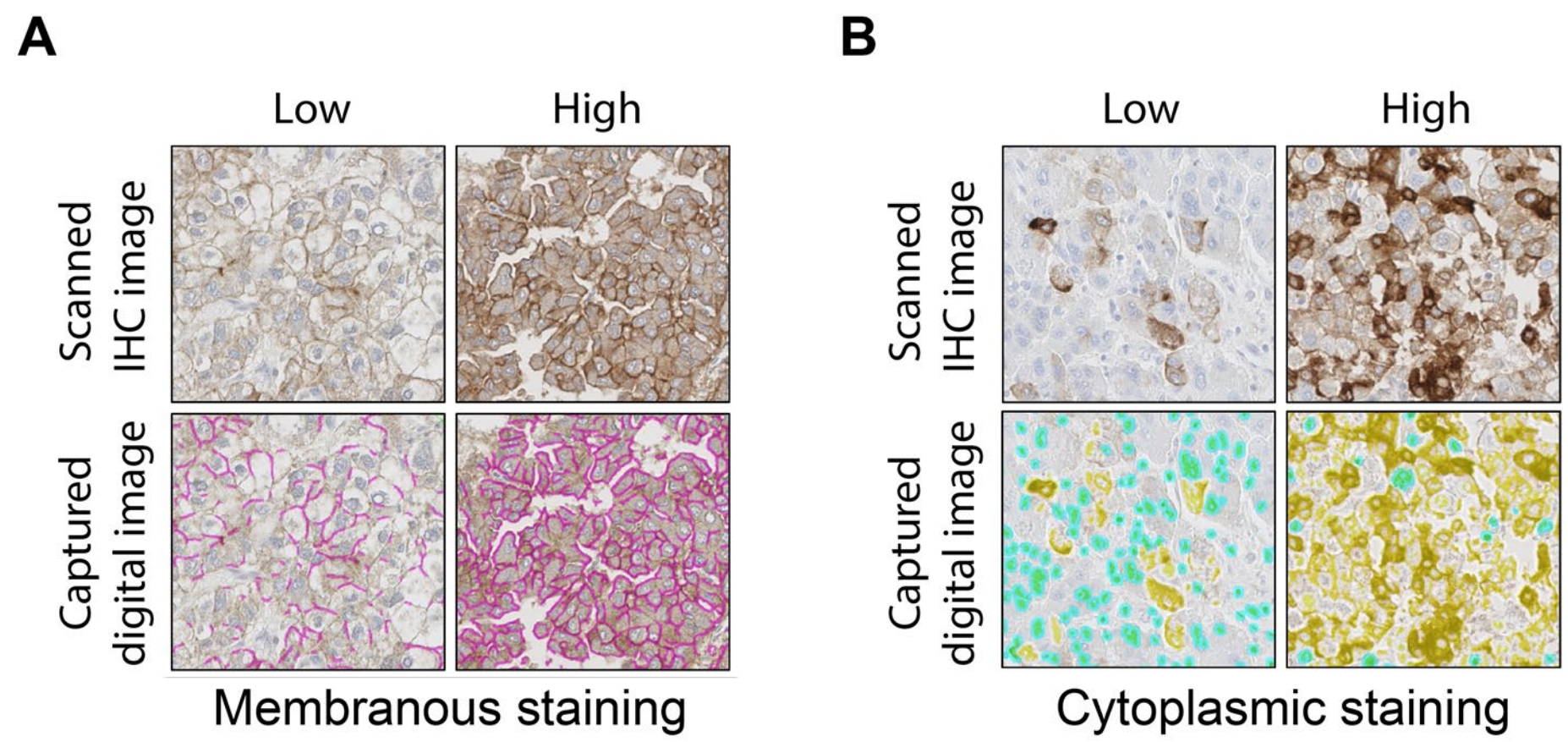

Figure S1. Visiopharm-based IHC scoring of membranous and cytoplasmic staining. (A) Representative scanned IHC images (top row) and corresponding Visiopharm-analyzed images (bottom row) showing low and high membranous expression. Membranous positivity is highlighted in pink, and nuclei are identified in cyan. **(B)** Representative scanned IHC images (top row) and corresponding Visiopharm-analyzed images (bottom row) showing low and high cytoplasmic expression. Positive cytoplasmic staining is segmented in yellow, and nuclei are identified in cyan.

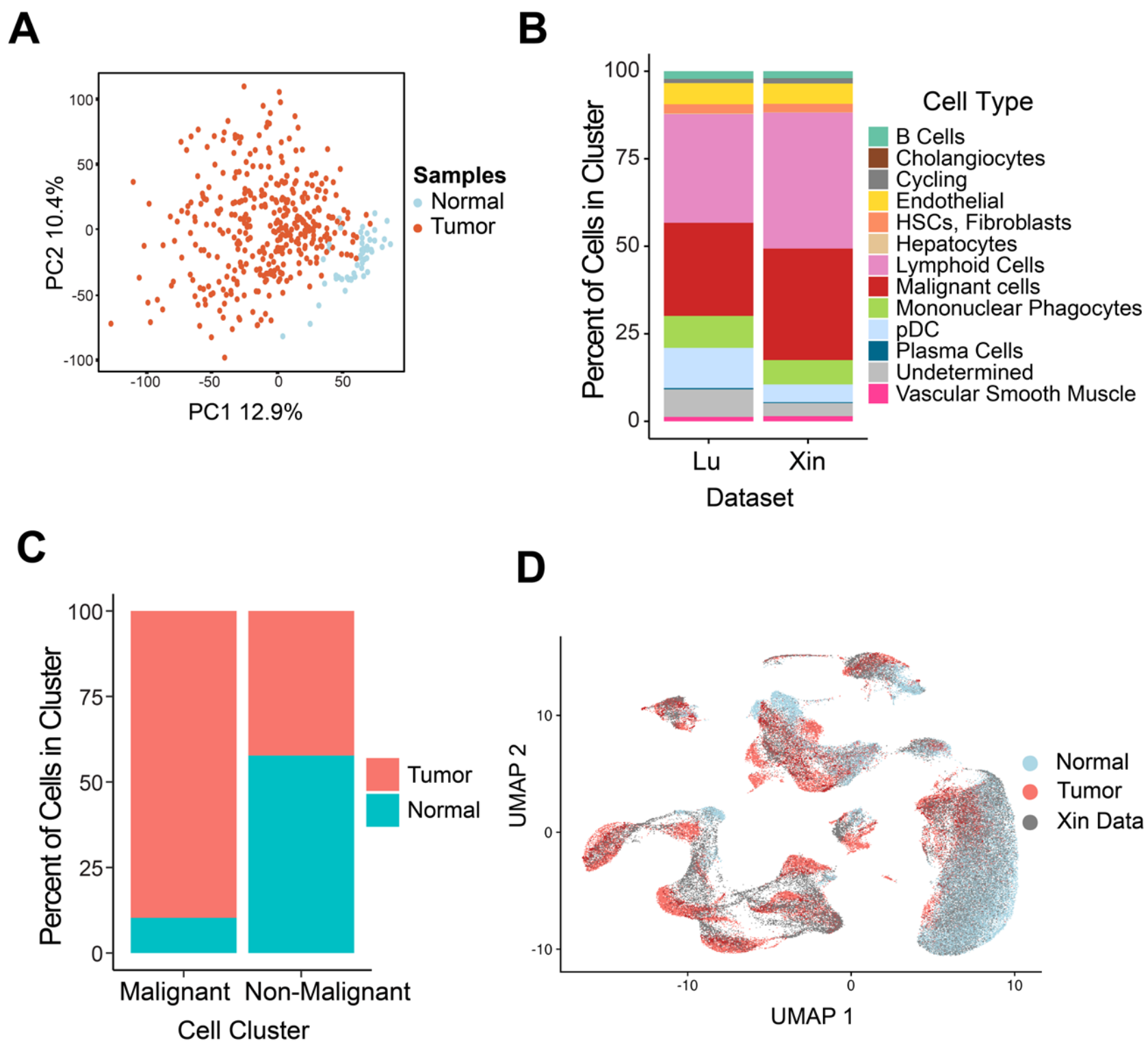

Figure S2. Integration of liver tissue Single Cell datasets. (**A**) PCA plot of bulk RNA seq data from TCGA-LIHC consisting of 371 HCC tumor (red) and 50 normal liver tissue (blue) samples. (**B**) Bar graph for proportion of major cell types in each single cell dataset. (**C**) Bar graph for proportion of malignant and non-malignant cells in each single cell dataset. (**D**) UMAP projection of all cells from integrated single cell datasets colored by normal (blue) and tumor (red) cells.

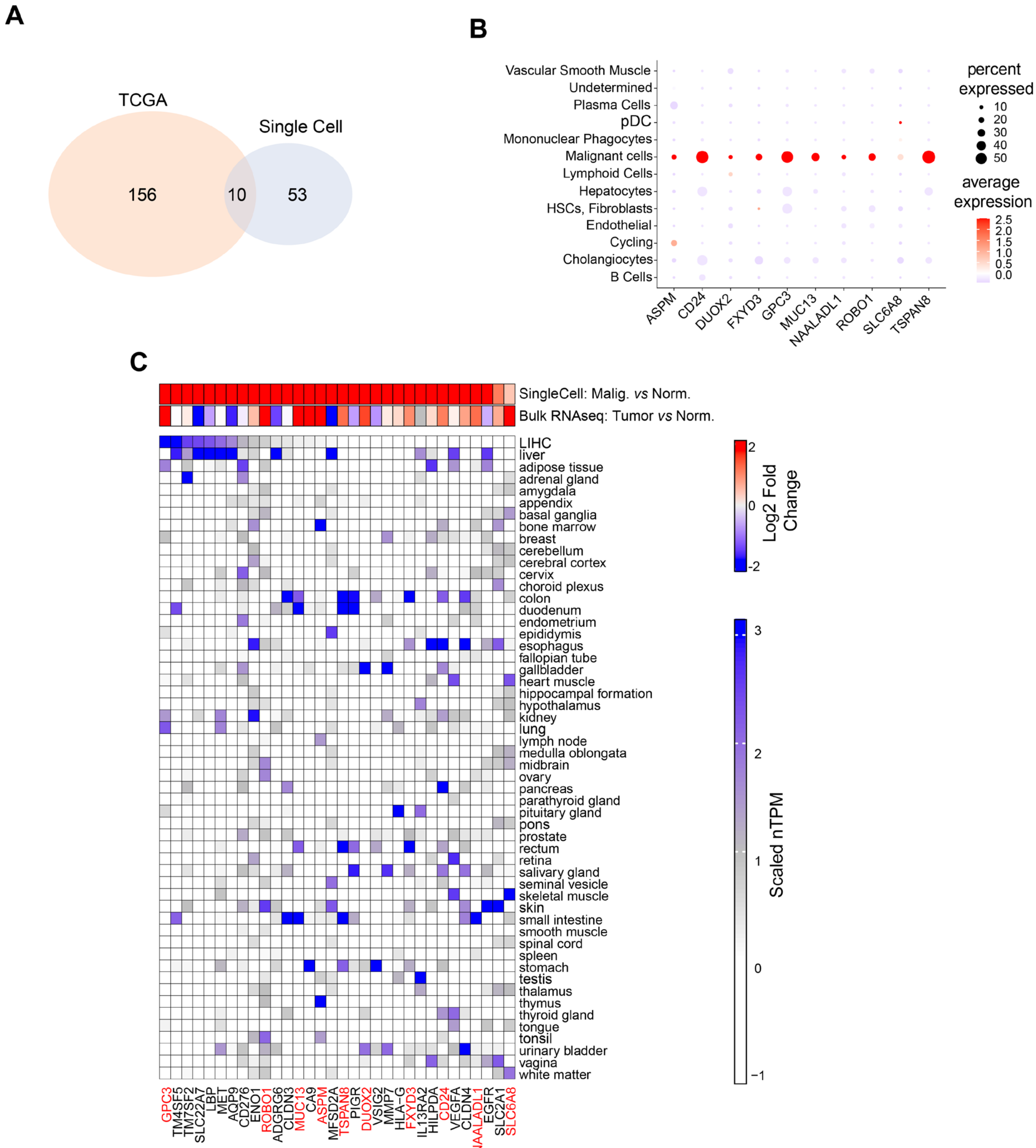

Figure S3. Identification and Exploration of HCC specific theranostic targets. (**A**) Venn Diagram comparing differentially expressed genes identified from the single cell data sets (blue - malignant vs non-malignant) and the TCGA-LIHC bulk RNA-seq data set (red – tumor vs normal). (**B**) Dot plot of differentially expressed genes identified in both the single cell and RNA-seq datasets for all major cell populations in the single cell data. The size of the circle indicates the percentage of cells expressing a given gene within the cell type and the color indicates the average expression of the gene (low – white, high – red) (**C**) Heatmap showing select differentially expressed malignant-cell-enriched genes from single cell

datasets. The main heatmap is colored by nTPM values for the consensus dataset and the TCGA cancer dataset (LIHC) from the Human Protein Atlas database (PADB). Gene expression values are normalized in each column using z-score scaling across tissues ranging from low (white) to high (dark blue) expression. The upper heatmap shows the $\log_2$ Fold Change values from the single cell dataset comparing malignant cells to normal cells and the $\log_2$ Fold Change (blue-decreased to red-increased) from TCGA-LIHC Bulk RNAseq analysis comparing tumor and normal samples.

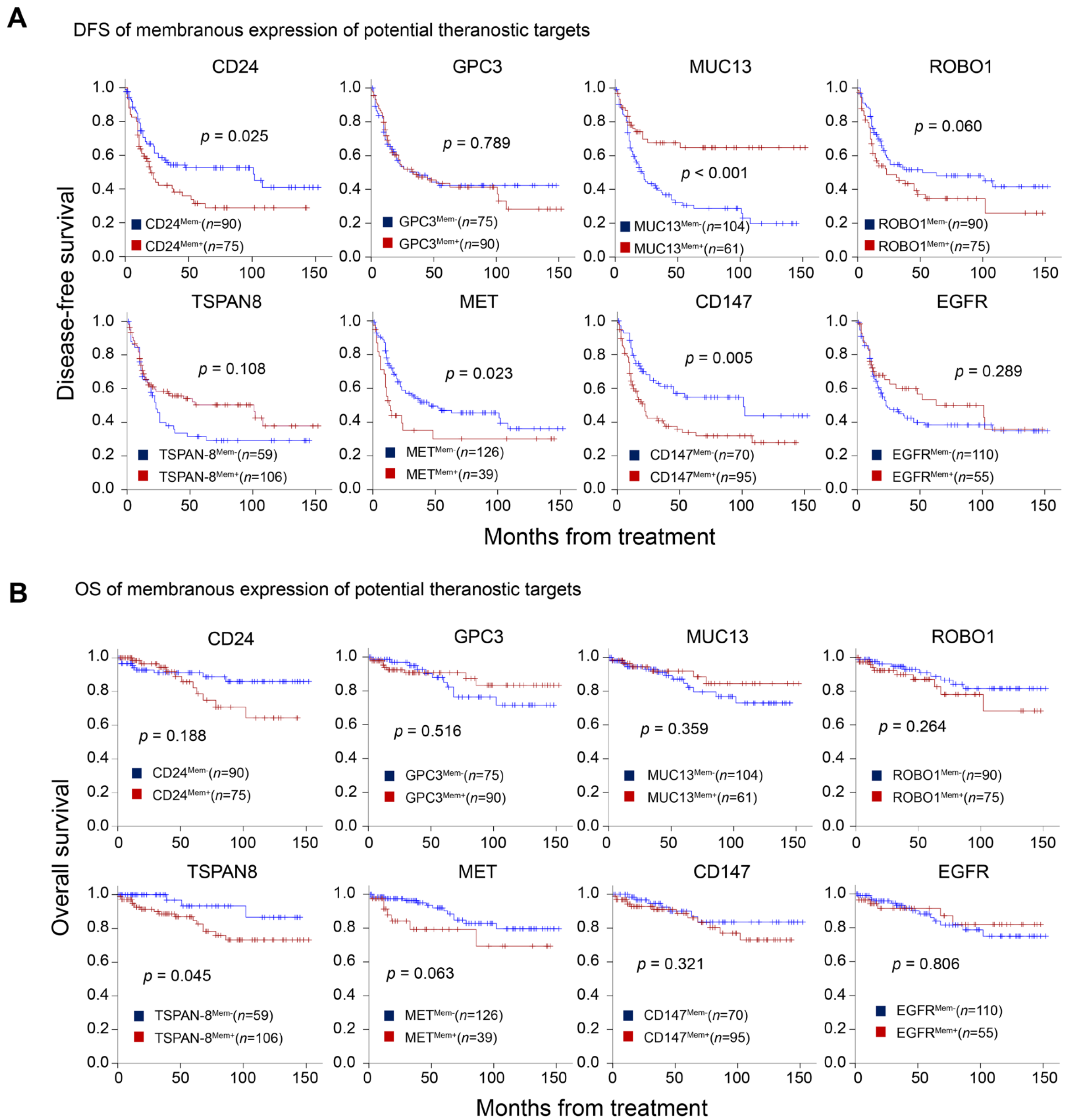

Figure S4. Survival analysis of HCC patients based on membranous expression of potential theranostic targets. Kaplan-Meier survival curves comparing disease-free survival (**A**) and overall survival (**B**) between groups classified by high and low membranous expression levels of theranostic targets. within the 165-patient cohort (individual n values for high/low groups are specified directly inside each panel). Statistical significance was evaluated using the log-rank test, with exact *p* values annotated on each curve.

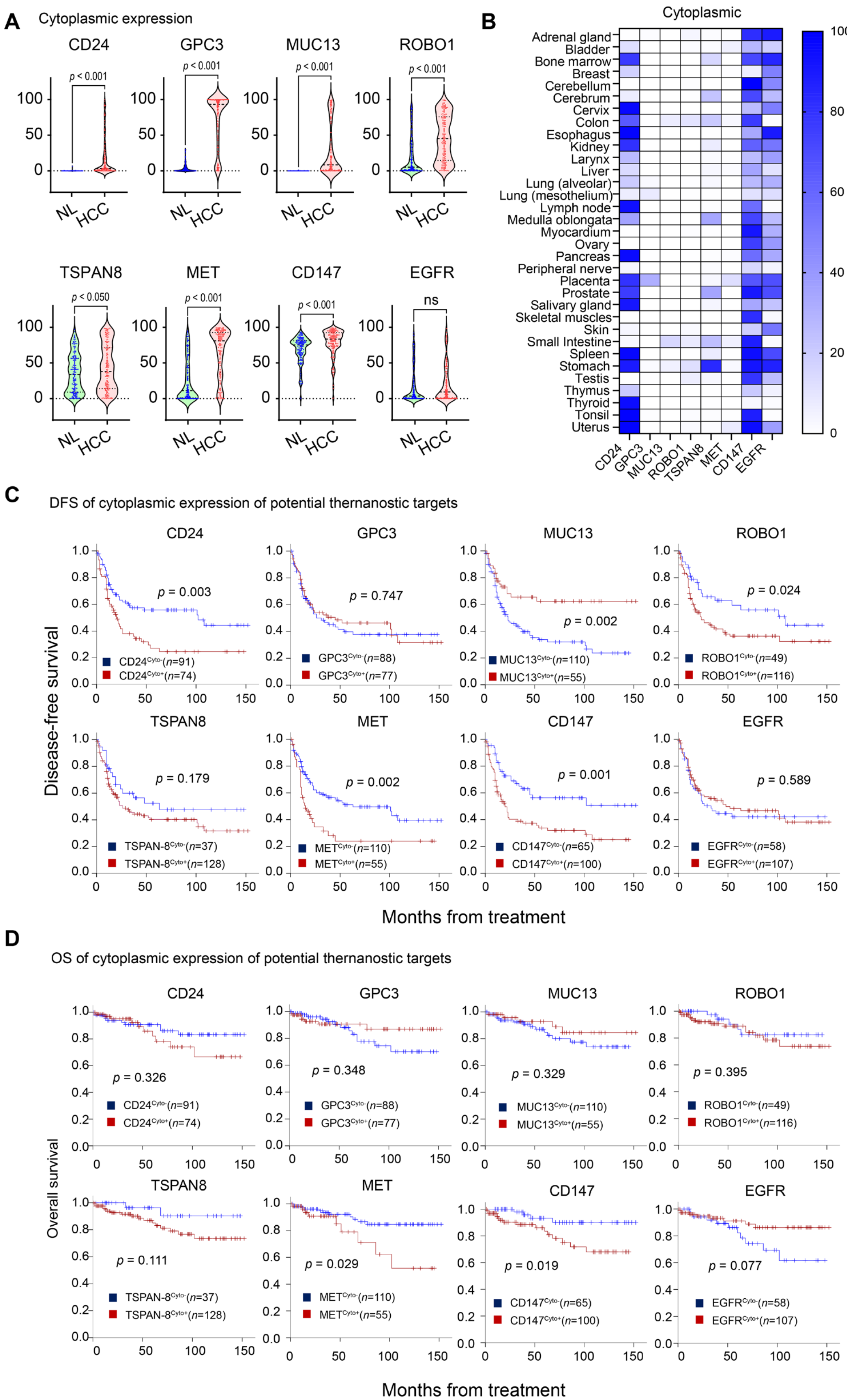
A
Cytoplasmic expression
CD24
GPC3
MUC13
ROBO1
TSPAN8
MET
CD147
EGFR
p < 0.001
p < 0.050
ns
NL
HCC
B
Cytoplasmic
Adrenal gland
Bladder
Bone marrow
Breast
Cerebellum
Cerebrum
Cervix
Colon
Esophagus
Kidney
Larynx
Liver
Lung (alveolar)
Lung (mesothelium)
Lymph node
Medulla oblongata
Myocardium
Ovary
Pancreas
Peripheral nerve
Placenta
Prostate
Salivary gland
Skeletal muscles
Skin
Small Intestine
Spleen
Stomach
Testis
Thymus
Thyroid
Tonsil
Uterus
C
DFS of cytoplasmic expression of potential thernanostic targets
Disease-free survival
p = 0.003
CD24Cyto- (n=91)
CD24Cyto+ (n=74)
p = 0.747
GPC3Cyto- (n=88)
GPC3Cyto+ (n=77)
p = 0.002
MUC13Cyto- (n=110)
MUC13Cyto+ (n=55)
p = 0.024
ROBO1Cyto- (n=49)
ROBO1Cyto+ (n=116)
p = 0.179
TSPAN-8Cyto- (n=37)
TSPAN-8Cyto+ (n=128)
p = 0.002
METCyto- (n=110)
METCyto+ (n=55)
p = 0.001
CD147Cyto- (n=65)
CD147Cyto+ (n=100)
p = 0.589
EGFRCyto- (n=58)
EGFRCyto+ (n=107)
Months from treatment
D
OS of cytoplasmic expression of potential thernanostic targets
Overall survival
p = 0.326
CD24Cyto- (n=91)
CD24Cyto+ (n=74)
p = 0.348
GPC3Cyto- (n=88)
GPC3Cyto+ (n=77)
p = 0.329
MUC13Cyto- (n=110)
MUC13Cyto+ (n=55)
p = 0.395
ROBO1Cyto- (n=49)
ROBO1Cyto+ (n=116)
p = 0.111
TSPAN-8Cyto- (n=37)
TSPAN-8Cyto+ (n=128)
p = 0.029
METCyto- (n=110)
METCyto+ (n=55)
p = 0.019
CD147Cyto- (n=65)
CD147Cyto+ (n=100)
p = 0.077
EGFRCyto- (n=58)
EGFRCyto+ (n=107)
Months from treatment

Figure S5. Clinical significance of cytoplasmic expression of potential theranostic markers in HCC patients. (**A**) Violin plots showing the distribution of theranostic markers based on cytoplasmic expression in HCC patient samples and adjacent non-tumor tissues (n = 165 pairs). Statistical analysis was performed using a two-tailed t-test. (**B**) Heatmap showing protein expression profiles in normal human tissues based on cytoplasmic localization (compiled from 33 distinct normal organs and 99 total cores). Kaplan-Meier plots for disease-free survival (**C**) and overall survival (**D**) based on cytoplasmic expression levels of potential theranostic markers in HCC patients (individual group n values are provided inside each plot).

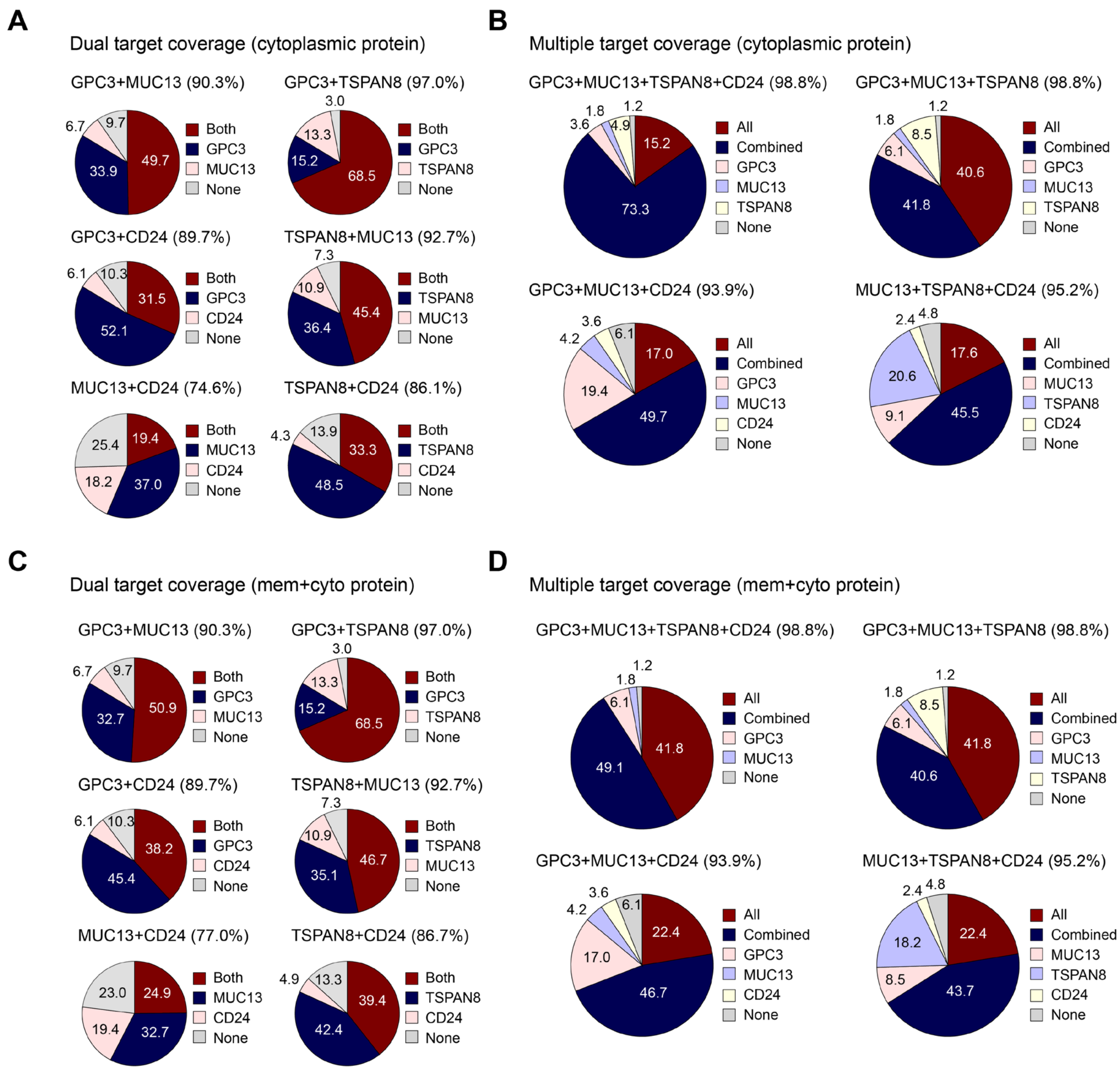

Figure S6. Distribution of potential theranostic marker expression in HCC based on cytoplasmic and combined membranous and cytoplasmic localization. The pie chart (**A**) represents the expression of dual targets in the cytoplasm, while chart (**C**) shows the combined membranous and cytoplasmic expression in the cohort of 165 HCC patients. The pie charts in (**B**) and (**D**) depict the expression profiles when multiple targets are combined, with coverage percentages indicated on each chart.

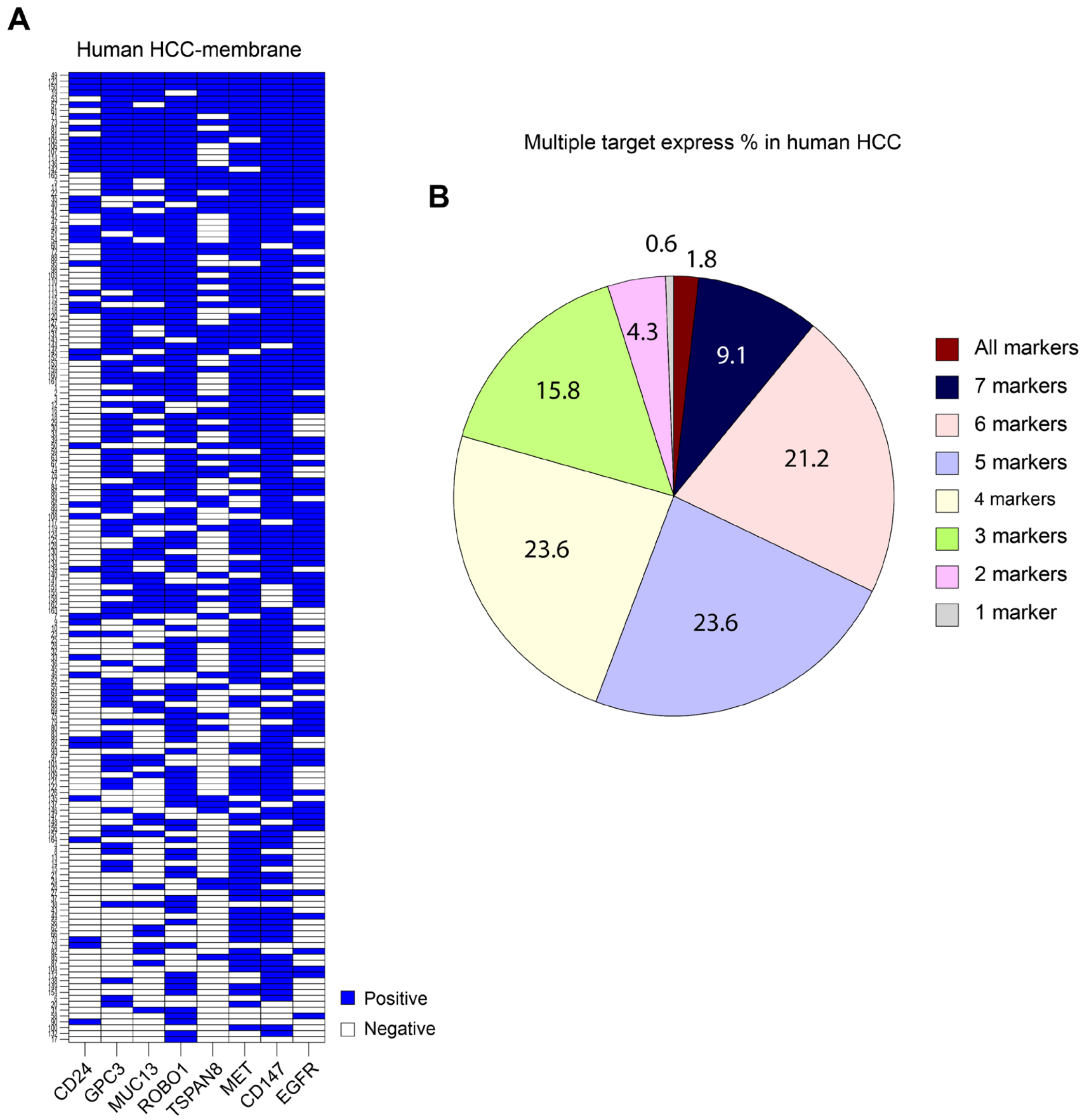

Figure S7. Membranous localization and expression patterns of candidate theranostic markers in HCC patient tissues. (**A**) Map illustrates immunohistochemically positive and negative staining profiles across the human HCC tissue cohort (n = 165). (**B**) Pie chart shows the proportions of distinct combinational theranostic markers expression patterns identified within the patient samples.

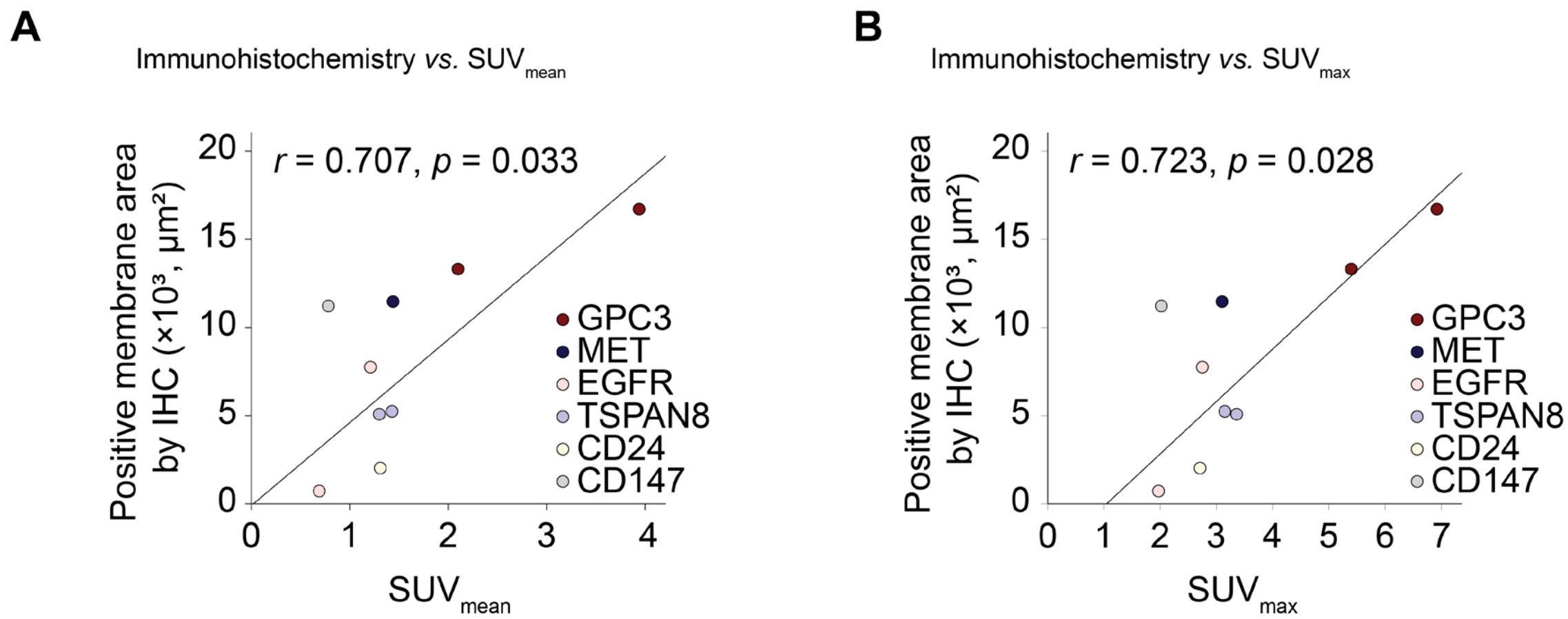

Figure S8. Correlation between immunohistochemical membrane staining and PET-derived standardized uptake values (SUVs) in HCC tissues. Quantitative analysis reveals a strong positive correlation between the extent of membranous immunohistochemical staining and both the mean of SUV (**A**, SUV_{mean}, $r = 0.707$, $p = 0.033$) and maximum of SUV (**B**, SUV_{max}, $r = 0.723$, $p = 0.028$). Statistical correlation strength and significance were calculated using Pearson's correlation coefficient profiling.

Table S2. List of antibodies for flow cytometry

Target	Isotype	Conjugate	Clone#/name	Vendor
CD24	Mouse IgG1κ	APC	SWA11	Cell Sciences
GPC3	Human IgG1κ	APC	Codrituzumab	Chugai Pharmaceutical
MUC13	Rabbit IgG	Unconjugated	EPR21901	Abcam
ROBO1	Rabbit IgG	Unconjugated	Polyclonal	Proteintech
TSPAN8	Rat IgG2b	Unconjugated	458811	R&D systems
MET	Human IgG1κ	PE	Onartuzumab	Genetech
CD147	Mouse IgG2b	APC	OTI4E4	OriGene
EGFR	Human IgG2κ	APC	Panitumumab	Amgen
Mouse IgG	Mouse IgG1	Unconjugated	CT6	Invitrogen
Rabbit IgG	Rabbit IgG	Unconjugated	EPR25A	Abcam
Human IgG	Human IgG	FITC	Polyclonal	Novus biologicals
Mouse IgG	Goat IgG	Alexa Fluor™ 647	Polyclonal	Invitrogen
Rabbit IgG	Goat IgG	Alexa Fluor™ 647	Polyclonal	Invitrogen
Rat IgG	Goat IgG	APC	Polyclonal	R&D systems

Table S3. Antibodies used for immunohistochemistry

Antibody	Vendor	Clone#	Cat. #	Incubation	Dilution	Antigen retrieval	Tissue
CD24	Cell Science	SWA11	FHD65210B	30 min at RT	1/2000	20 min, PC, pH 6	Human TMA
GPC3	Cell Marque	1G12	261M-96	60 min at RT	1/500	20 min, PC, pH 6	Human TMA
MUC13	Cell Signaling	E6Z1K	44454	60 min at RT	1/200	20 min, PC, pH 6	Human TMA
ROBO1	Proteintech	Monoclonal	67922-1-Ig	30 min at RT	1/2000	20 min, PC, pH 9	Human TMA
TSPAN8	Abcam	Polyclonal	ab7007	30 min at RT	1/5000	20 min, PC, pH 6	Human TMA
MET	Cell Signaling	D1C2	8198	60 min at RT	1/500	20 min, PC, pH 9	Human TMA
CD147	Proteintech	Monoclonal	66443-1-Ig	60 min at RT	1/250	20 min, PC, pH 6	Human TMA
EGFR	Dako	H11	M356301-2	60 min at RT	1/150	Pro-K, 5 min at RT	Human TMA
CD24	Invitrogen	SN3	MA5-11828	30 min at RT	1/200	20 min, PC, pH 6	Xenograft
GPC3	Abcam	SP86	ab95363	30 min at RT	1/3000	20 min, PC, pH 6	Xenograft
TSPAN8	Abcam	Polyclonal	ab70007	30 min at RT	1/1000	20 min, PC, pH 6	Xenograft
MET	Cell Signaling	D1C2	8198	60 min at RT	1/200	20 min, PC, pH 9	Xenograft
CD147	Thermo Fisher Scientific	125	MA5-29060	30 min at RT	1/5000	20 min, PC, pH 6	Xenograft
EGFR	Cell Signaling	D38B1	4267	60 min at RT	1/100	20 min, PC, pH 9	Xenograft
Ki-67	Abcam	SP6	ab16667	60 min at RT	1/200	20 min, PC, pH 6	Xenograft

RT: room temperature; PC: pressure cooker; Pro-K: proteinase-K

Table S4. Quality control parameters and radiochemical profiles of the evaluated zirconium-89 labeled antibodies and fluorine-18 labeled nanobody.

**Tracer**	**RCY (%)**	**Radiochemical purity (%)**	**Specific activity**	**Molar activity**	**n**
[^{89}Zr]Zr-GC33	90–95	>98	373–1,400 MBq/mg	56–210 GBq/µmol	26
[^{89}Zr]Zr-Pani	77–93	>98	147–287 MBq/mg	22–43 GBq/µmol	5
[^{89}Zr]Zr-DFO-α-hCD24	70–80	>98	227–280 MBq/mg	34–42 GBq/µmol	3
[^{89}Zr]Zr-DFO-α-hTSPAN8	71–83	>98	200–253 MBq/mg	30–38 GBq/µmol	2
[^{18}F]FPY-DMN1	12–18	>98	247–740 GBq/mg	4–11 TBq/µmol	5

RCY: radiochemical yield; GBq: gigabecquerel; TBq: terabecquerel; MBq: megabecquerel; n: total number of independent production batches. Data are presented as ranges reflecting minimum and maximum values obtained across all successful synthesis runs.

Table S5. Cox proportional univariate and multivariate analyses of disease-free survival and overall survival in patients with HCC

**Variables**	**Disease-free survival**				**Overall survival**			
	Univariate analysis		Multivariate analysis		Univariate analysis		Multivariate analysis	
	HR [95% CI]	*p* value	HR [95% CI]	*p* value	HR [95% CI]	*p* value	HR [95% CI]	*p* value
Age (>60)	0.975 [0.635-1.497]	0.907			1.454 [0.600-3.524]	0.407		
Sex (male)	1.673 [0.942-2.973]	0.079			0.934 [0.338-2.582]	0.895		
BMI (≥25)	0.886 [0.571-1.375]	0.591			0.560 [0.203-1.542]	0.262		
HBsAg+	0.880 [0.558-1.388]	0.582			0.503 [0.204-1.242]	0.136		
pT	1.578 [1.197-2.082]	0.001*	1.769 [1.306-2.396]	<0.001*	2.203 [1.266-3.833]	0.005*	2.546 [1.427-4.543]	0.002*
M stage (M1)	2.267 [1.092-4.704]	0.028*	2.697 [1.222-5.953]	0.014*	1.657 [0.382-7.186]	0.500		
CD147^{Mem}+	1.876 [1.203-2.923]	0.006*	1.056 [0.635-1.758]	0.833	1.563 [0.623-3.921]	0.341		
CD147^{Cyto}+	2.132 [1.344-3.380]	0.001*	1.866 [1.067-3.264]	0.029*	3.459 [1.155-10.364]	0.027*	4.250 [1.397-12.925]	0.011*
EGFR^{Mem}+	0.779 [0.489-1.239]	0.292			0.902 [0.346-2.349]	0.832		
EGFR^{Cyto}+	0.886 [0.574-1.367]	0.583			0.465 [0.19-1.125]	0.090		
GPC3^{Mem}+	1.054 [0.689-1.610]	0.809			0.758 [0.314-1.829]	0.537		
GPC3^{Cyto}+	0.927 [0.608-1.413]	0.724			0.657 [0.262-1.646]	0.370		
MET^{Mem}+	1.726 [1.075-2.771]	0.024*	1.586 [0.908-2.769]	0.105	2.316 [0.920-5.826]	0.075		
MET^{Cyto}+	1.841 [1.202-2.820]	0.005*	1.166 [0.691-1.967]	0.566	2.004 [0.826-4.862]	0.125		
TSPAN8^{Mem}+	0.708 [0.463-1.081]	0.110			3.283 [0.962-11.209]	0.058		
TSPAN8^{Cyto}+	1.429 [0.841-2.429]	0.187			3.118 [0.723-13.442]	0.127		
MUC13^{Mem}+	0.386 [0.231-0.643]	<0.001*	0.306 [0.044-2.137]	0.232	0.649 [0.249-1.691]	0.376		
MUC13^{Cyto}+	0.445 [0.265-0.749]	0.002*	1.199 [0.166-8.667]	0.857	0.615 [0.223-1.695]	0.347		
ROBO1^{Mem}+	1.492 [0.978-2.276]	0.064			1.684 [0.695-4.081]	0.249		
ROBO1^{Cyto}+	1.773 [1.074-2.926]	0.025*	2.519 [1.468-4.322]	0.001*	1.568 [0.568-4.330]	0.385		
CD24^{Mem}+	1.620 [1.061-2.472]	0.025*	0.864 [0.481-1.550]	0.623	1.775 [0.732-4.304]	0.205		
CD24^{Cyto}+	1.915 [1.252-2.930]	0.003*	1.799 [1.023-3.164]	0.041*	1.529 [0.634-3.692]	0.345		

HR: Hazard ratio; CI: confidence interval; HBsAg: Hepatitis B surface antigen

*Statistically significant ($p < 0.05$)
